# Dynactin1 depletion leads to neuromuscular synapse instability and functional abnormalities

**DOI:** 10.1186/s13024-019-0327-3

**Published:** 2019-07-10

**Authors:** Valérie Bercier, Jeffrey M. Hubbard, Kevin Fidelin, Karine Duroure, Thomas O. Auer, Céline Revenu, Claire Wyart, Filippo Del Bene

**Affiliations:** 1 Institut Curie, PSL Research University, INSERM U934, CNRS UMR3215, Sorbonne Université, F-75005 Paris, France; 2 Sorbonne Université, Inserm, CNRS, AP-HP, Institut du Cerveau et de la Moelle Épinière, ICM, F-75013 Paris, France; 3 Present Address: VIB-KU Leuven, Center for Brain & Disease Research, Leuven, Belgium; 4 Present Address: Zuckerman Mind Brain Behavior Institute, Columbia University, New York, USA; 5 Present Address: Center for Integrative Genomics, University of Lausanne, Lausanne, Switzerland

**Keywords:** Dynactin1, Zebrafish, Neuromuscular junction, Amyotrophic lateral sclerosis, Axonal transport

## Abstract

**Background:**

Dynactin subunit 1 is the largest subunit of the dynactin complex, an activator of the molecular motor protein complex dynein. Reduced levels of *DCTN1* mRNA and protein have been found in sporadic amyotrophic lateral sclerosis (ALS) patients, and mutations have been associated with disease, but the role of this protein in disease pathogenesis is still unknown.

**Methods:**

We characterized a Dynactin1a depletion model in the zebrafish embryo and combined in vivo molecular analysis of primary motor neuron development with live in vivo axonal transport assays in single cells to investigate ALS-related defects. To probe neuromuscular junction (NMJ) function and organization we performed paired motor neuron-muscle electrophysiological recordings and GCaMP calcium imaging in live, intact larvae, and the synapse structure was investigated by electron microscopy.

**Results:**

Here we show that Dynactin1a depletion is sufficient to induce defects in the development of spinal cord motor neurons and in the function of the NMJ. We observe synapse instability, impaired growth of primary motor neurons, and higher failure rates of action potentials at the NMJ. In addition, the embryos display locomotion defects consistent with NMJ dysfunction. Rescue of the observed phenotype by overexpression of wild-type human DCTN1-GFP indicates a cell-autonomous mechanism. Synaptic accumulation of DCTN1-GFP, as well as ultrastructural analysis of NMJ synapses exhibiting wider synaptic clefts, support a local role for Dynactin1a in synaptic function. Furthermore, live in vivo analysis of axonal transport and cytoskeleton dynamics in primary motor neurons show that the phenotype reported here is independent of modulation of these processes.

**Conclusions:**

Our study reveals a novel role for Dynactin1 in ALS pathogenesis, where it acts cell-autonomously to promote motor neuron synapse stability independently of dynein-mediated axonal transport.

**Electronic supplementary material:**

The online version of this article (10.1186/s13024-019-0327-3) contains supplementary material, which is available to authorized users.

## Background

ALS is an adult-onset, neurodegenerative disease affecting upper and lower motor neurons. It leads to denervation at the neuromuscular junction, muscle wasting and progressive paralysis, ending in the demise of the patient two to 5 years after diagnosis. Many causative mutations and risk factors have been identified in the last few decades targeting genes like *SOD1, TARDBP* and *FUS,* and more recently, *C9ORF72,* along with multiple pathogenic mechanisms, including protein misfolding and aggregation, impaired RNA metabolism and excitotoxicity (reviewed here [1–5]). Recent evidence revealed multiple cases where an inheritance of risk variants in multiple genes led to disease or altered penetrance of pathogenic mutations. Considering the sporadic nature of most cases, this proposed oligogenic etiology of ALS [4, 6–8] highlights the importance of studies focused on rare variants. Indeed, as ALS remains a disease of heterogeneous clinical presentation and variable progression, diagnosed on exclusion, insights into pathophysiological processes could help categorization and lead to the elaboration of effective treatment targeting specific mechanisms.

Axonal transport defects is a common mechanism that has been observed in many neurodegenerative diseases, including ALS [9–11]. Indeed, defects in both fast and slow axonal transport have been reported for cargoes such as mitochondria, endosomes and vesicles containing trophic signaling receptors like TrkB [12–16]. As motor neurons extend long projections to reach their target, they are particularly dependent on axonal transport for communication between the synapse and the cell body, to supply the synapse with building components and to remove cellular detritus for degradation. Axonal transport is mediated by ATP-driven molecular motors, which carry vesicles and organelles by moving along the microtubule network. This component of the cell cytoskeleton is composed of protofilaments polymerized from tubulin subunits which are oriented in a highly polarized manner in the axon, resulting in a fast-growing end (+) at the synapse and a slow-growing end (−) oriented toward the nucleus at the soma. The dynein motor complex transports cargo in the retrograde direction (− end directed) [17] and relies on multi-subunit complexes like dynactin for functional versatility [18, 19].

Dynactin subunit 1 (*DCTN1*, ENSG00000204843; OMIM 601143, hereafter referred to as Dynactin1) is the largest subunit of the dynactin complex and acts as the link between this macromolecular complex and the microtubules via its CAP-Gly domain [20, 21]. The dynactin complex regulates the activity of molecular motor complex dynein, where Dynactin1 serves as the link between both complexes [20, 22], and orchestrates the motor’s recruitment to the microtubule network at distal ends [20, 23]. It has therefore been thought to be essential for dynein function, and for axonal transport [24]. *DCTN1* was found to be down regulated in sporadic ALS patients, resulting in a lower protein expression [25, 26], suggesting altered expression of this protein could be involved in the pathophysiological process.

Mutations in *DCTN1* have also been reported in ALS patients [8, 27–31], however their role in motor neuron degeneration is not clear. Indeed, mutations in this gene have previously been found in patients affected with Perry Syndrome, a disease characterized by Parkinsonism. In this case, the reported mutations affect the the N-terminal CAP-Gly domain (G59S) interfere with the incorporation of this subunit within the dynactin complex and leads to the formation of misfolded protein aggregates in a phenotype similar to what is observed following the loss of the whole CAP-Gly domain [32, 33]. In contrast, the mutations reported in ALS cases don’t target a particular domain (for instance T1249I, M571 T, R785W, R1101K [28], I196V and R1049Q [30]) and have been found to lead to proper incorporation of Dynactin1 within the dynactin complex without aggregate formation (for M571 T, R785W, R1101K and T1249I [34]). Because of its known function in regulating dynein activity and because axonal transport deficits are a feature of ALS, Dynactin1’s implication in neurodegeneration has been assumed to involve axonal transport dysregulation [5, 35].

Here, we analyze the effect of Dynactin1 depletion in the zebrafish embryo (genetic inactivation ofortholog *dynactin1a* ENSDARG00000019743; mutant line mok^m632^) on the development of the caudal primary (CaP) motor neurons of the spinal cord. Homozygous mutant embryos, referred to as mok ^m632−/−^ hereafter, initially extend normal CaPs and establish proper neuromuscular junctions (NMJ) with fast-twitch muscle fibers. Depletion of Dynactin1a leads to synapse instability, which impairs further growth and causes electrophysiological dysfunction and locomotor deficits. These defects were found to be independent of changes in axonal transport dynamics or cytoskeletal modulation, two well-known functions relying on Dynactin1. Our results point to a local role for this protein in synapse stability where a protein depletion could contribute to the oligogenic etiology of ALS pathogenesis by inducing NMJ dysfunction without leading to ALS-related motor neuron degeneration in itself.

## Material and methods

### Zebrafish husbandry and transgenic lines

Zebrafish lines were housed in the Curie Institute animal facility, maintained at 28,5 °C and embryos were raised in egg medium containing methylene blue. All experiments were performed according to the French and European Union animal welfare guidelines, as well as the Curie Institute ethics protocol.

The following transgenic and mutant fish lines were used: *Tg(mnx1:Gal4)* [36]*; mok* ^*m632*^*(dctn1a*^*m632/m632*^*)* [37–39]*; Tg(UAS:GCaMP5G)* [40]; *Tg(cdh2:Cdh2-GFP)* [41].

### Molecular cloning

*4nrUAS-tagRFPCaax-pA-4nrUAS-eGFP-Rab5c-pA;cmcl2:eGFP;*

*-Rab7-pA;cmcl2:eGFP;*

*-Rab11a-pA;cmcl2:eGFP*

Fusion proteins were generated by fusing *rab5c*, and *rab7* open reading frames from p3’E vectors (kindly provided by Brian Link [42]) with eGFP into pME (Tol2Kit) [43]. UAS constructs were assembled by combining pME-rab-GFP, p3’E-*SV40* and a *p5′E-4nrUAS-tagRFPCaax-pA-4nrUAS* vector (containing a membrane-bound *tagRFP* reporter under the expression of four non-repeated UAS sequences) [44] into a pDestTol2CG #393 destination vector [43] using the MultiSite Gateway Three-Fragment Vector Construction Kit (ThermoFisher Scientific). The *rab11a* cDNA was amplified from zebrafish total cDNA using primers 5’E- atggggacacgagacgacg and 5′- ctagatgctctggcagcactg and cloned into a pDONRP2R-P3 to generate a p3’E vector, which was combined with a pME-eGFP vector and a *p5’-4nrUAS-tagRFPCaax-pA-4nrUAS* vector into a pDestTol2CG #393 destination vector [43] using the MultiSite Gateway Three-Fragment Vector Construction Kit (ThermoFisher Scientific).

#### mnx1:lyn-GFP-pA

A p5’E entry vector was generated by PCR amplification of a 125 bp promoter fragment of the *mnx1* gene [36] followed by a BP reaction. The middle entry plasmid was obtained by BP reaction from amplification of two consecutive copies of a sequence encoding the *Palm-myr* signal of Lyn kinase from *Mus musculus* (MGCIKSKRKDNLNDDE). The construct was assembled into a pDONR221 using the MultiSite Gateway Three-Fragment Vector Construction Kit (ThermoFisher Scientific) to obtain *mnx1:lyn-eGFP-pA*.

#### pUAS-dendra2-rab3-pA

A middle entry vector carrying *dendra2-rab3* was generated by fusing the *rab3* open reading frame of *pBHUAS-Rab3-YFP* (kindly provided by Michael Nonet) [45], and the *dendra2* sequence from *pDendra2-N1* (kindly provided by Jean-René Huynh, Institut Curie, Paris) via PCR amplification. The middle entry vector was combined with a standard *p5’UAS* vector (Tol2kit) and a standard *p3’SV40pA* using the MultiSite Gateway Three-Fragment Vector Construction Kit (ThermoFisher Scientific) to obtain *pUAS-dendra2-rab3-pApUAS-dendra2-rab3-pA.*

#### pUAS-EB3-meGFP-pA

The *pME-EB3* plasmid [41] was combined with *p3’meGFP* under a *UAS* promoter and assembled into pDONR221 using the MultiSite Gateway Three-Fragment Vector Construction Kit (ThermoFisher Scientific) to obtain *pUAS-EB3-GFP.*

#### 14xUAS:ubc-EB3-meGFP-E2A-tagRFP-rab3-pA

This construct was obtained via Gibson assembly using the *pT1UciMP Tol1* destination vector previously described. The *EB3-meGFP* fragment was amplified via PCR from *pUAS-EB3-meGFP-pA*, the *E2A-tagRFP* was amplified via PCR from *4nrUAS-tagRFPCaax-pA-4nrUAS-eGFP-Rab5c-pA,* and the *rab3-pA* was amplified via PCR from *pUAS-dendra2-rab3-pA.* All fragments were inserted after the *ubc* intron of the *pT1UciMP Tol1* destination vector opened by restriction digest with NcoI-HF (NEB) to obtain *14xUAS:ubc-EB3-meGFP-E2A-tagRFP-rab3-pA.*

#### 14xUAS:ubc-hDCTN1-eGFP-E2A-tagRFPCaax-pA

This construct was obtained via Gibson assembly using the *pT1UciMP Tol1* destination vector previously described. *Dynactin1* was amplified via PCR from a pCDNA3.1 vector containing human *Dynactin1-*GFP (kindly provided by Stefan Liebau [30]) and fused to *eGFP* and E2A-tagRFPCaax (described above) using the NEBuilder HiFi DNA Assembly Cloning Kit (NEB). These three sequences were inserted after the *ubc* intron of the *pT1UciMP Tol1* destination vector opened by restriction digest with NcoI-HF (NEB) to obtain *14xUAS:ubc-hDCTN1-eGFP-E2A-tagRFPCaax-pA.*

#### 14xUAS:ubc-ngfra-eGFP-E2A-tagRFPCaax-pA

Similar to the construct above, the cDNA sequence of the *ngfra* zebrafish gene (ENSDARG00000088708) coding for the p75 trophic receptor was amplified from zebrafish cDNA via PCR and combined with *eGFP* and E2A-tagRFPCaax into the *pT1UciMP Tol1* destination vector using the NEBuilder HiFi DNA Assembly Cloning Kit (NEB) to obtain *14xUAS:ubc-ngfra-eGFP-E2A-tagRFPCaax-pA.*

### RNA synthesis

RNA for human Dynactin1-GFP was synthesized from the pCDNA3.1 construct described previously (kindly provided by Stefan Liebau [30]) using the mMESSAGE mMACHINE T7 transcription kit (Invitrogen).

### Microinjections

Embryos were injected at the zygote stage (1 cell) using a Picospritzer III pressure ejector and a glass capillary tube pulled with a Flaming-Brown puller as a needle. Injection mixes contained phenol red to judge injected volume and were set to 400 ng/ul of RNA, and 30-50 ng/ul of recombinant DNA with or without added transposase mRNA (50 ng/ul).

### Whole-mount immunohistochemistry

Embryos were fixed in 4% paraformaldehyde diluted in PBS for 4 h at room temperature. They were then rinsed multiple times in PBS containing 0,1% triton X-100 (PBST) then incubated with a solution of 1 mg/ml of collagenase (from Clostridium histolyticum, Sigma) in PBS for 20 min (2dpf embryos) or 2 h (6dpf embryos). The embryos were rinsed several times with PBST then blocked for 1 h in a block solution containing 1% bovine serum albumin (BSA), 2% normal goat serum, 1% DMSO and 0,1% triton X-100. The primary antibody was then added with fresh block solution according to the working dilutions listed below, with an incubation time of 2 h at room temperature. After several washes in PBST, the secondary antibody was added in fresh block solution for an incubation of 2 h at room temperature, then rinsed thoroughly. The embryos were then processed for imaging.

For labelling with conjugated α-bungarotoxin, the fixation step was done overnight and the block solution used was composed of 2% BSA, 0,5% triton X-100 in PBS. The incubation time for the conjugated α-bungarotoxin was 30 min at room temperature.

### List of antibodies

- Anti-synaptotagmin2 (znp1) (Developmental Studies Hybridoma Bank), monoclonal mouse IgG2a, used at 1:300.
- Conjugated α-bungarotoxin-AlexaFluor 594 (ThermoFischer Scientific), α-subunit of the nicotinic acetylcholine receptor (AChR) extracted from *Bungarus multicinctus* venom and conjugated with Alexa Fluor 594 used at 10μg/ml.
- Anti-acetylated tubulin clone 6–11-B-1 (Sigma) purified mouse monoclonal IgG antibody 1,5 mg/ml used at 1:200.
- Anti-GFP (GeneTex, Euromedex) purified chicken polyclonal IgG antibody, 10mg1ml, used at 1:300.
- Goat anti-chicken Alexa Fluor 488 (Life Technologies) purified goat antibody, used at 1:1000
- Goat anti-mouse Alexa Fluor 488 (Life Technologies) purified goat antibody, used at 1:1000
- Goat anti-mouse Alexa Fluor 635 (Life Technologies) purified goat antibody, used at 1:1000

### Touch-evoked escape response assay

2dpf embryos were dechorionated and left to acclimate at room temperature 30 min prior to the experiment. Each embryo was placed in the center of a 144 mm petri dish containing egg medium. A refractory period of 30 s was observed before the presentation of a stimulus. The escape response was elicited by a light brush on the tail of the embryo with a pair of blunt forceps and was recorded with an Olympus FE-5000 camera or with a Sony HDR-AS50 at 30 Hz. The videos were analyzed in ImageJ using the Manual Tracking plugin (Fabrice Cordelières, Institut Curie-Orsay, France).

### Morphological images

Images of the embryos were acquired with a Leica MZ FLIII stereomicroscope (Leica) equipped with a Leica DFC310FX digital camera (Leica).

### Fluorescence microscopy for RNA injection validation

Images of the GFP signal in the 488 nm wavelength channel was acquired on a Leica DM 3000 LED microscope equipped with a DMK 33UX250 USB3.0 monochrome industrial camera (The Imaging Source, Bremen, Germany) using Lucia 4.60 software (Laboratory Imaging, Prague, Czech Republic).

### Spinning disk confocal microscopy for cell morphology and time-lapse imaging

We limited our study to CaP motor neurons within a 4-somite window around the cloaca in order to avoid morphological and functional variability that arise between cell types and along the rostro-caudal developmental wave.

Imaging was performed on a Roper confocal spinning disk head mounted on a Zeiss upright microscope, and acquisitions were done with a CoolSNAP HQ2 CDD camera (Photometrics, USA) through the MetaMorph software (Molecular Devices, USA). Embryos were anesthetized using 0.02% tricaine (MS-222, Sigma) diluted in egg water and embedded in 1% low melting-point agarose in a glass-bottom cell tissue culture dish (Fluorodish, World Precision Instruments, USA). Acquisitions were done using water immersion long working distance lenses, at 40x magnification (W DIC PL APO VIS-IR; 421,462–9900) for z-stack images of the whole tectum and at 63x magnification (W PL APO VIS-IR (421480–9900) for single plane time-lapse imaging of linear axonal segments, and for filopodia imaging. Acquisitions were done using the Metamorph software (Molecular Devices) and resolution in z was set at 1um for stacks. Images were assembled and analyzed in ImageJ (NIH). 6dpf z- stacks taken in two frames were stitched together using the pairwise stitching function of the Stitching plugin [46].

### Time-lapse imaging

Live imaging of axonal transport was done using fusion proteins combined with a membrane reporter, described previously, expressed in the CaP primary motor neurons by use of the Tg(mnx1:GAL4) line. Time-lapse parameters were determined based on the speed of transport in the spinal cord and set at 1 s intervals for mitochondria (4nrUAS:tagRFPCaax-pA-4nrUAS:PhbGFP-pA-Tol2;cmcl2:eGFP), for 10 min total duration, and set at 500 ms for endosomes (rab5c, 7 and 11a), p75, and eb3 comets for 5 min total duration. For filopodia dynamics time-lapses, z-stacks were taken every 2 min for 10 min of total duration.

### Kymogram production and analysis

Time-lapse images were assembled and analyzed in ImageJ. Kymograms were extracted for each time-lapse serie on linear axonal segments using the Kymograph Tool (Montpellier RIO Imaging, CNRS, France), where each pixel on the Y axis represents one timepoint projected against axonal length (X axis).

### Calcium imaging during fictive locomotion

4dpf Tg(mnx1:gal4; UAS:GCaMP5G) double transgenic larvae were screened for dense labeling and good expression of GCaMP5 in spinal motor neurons under a dissecting microscope equipped with an epifluorescence lamp (Leica, Wetzlar, Germany). Larvae were anaesthetized in 0.02% Tricaine-Methiodide (MS-222, Sigma-Aldrich) diluted in fish facility water and mounted on their lateral side in 1.5% low-melting point agarose in glass-bottom dishes filled with external solution ([NaCl] = 134 mM, [KCl] = 2.9 mM, [MgCl2] = 1.2 mM, [HEPES] = 10 mM, [glucose] = 10 mM and [CaCl2] = 2.1 mM; adjusted to pH 7.7–7.8 with NaOH and osmolarity 290 mOsm). Larvae were immobilized by injecting 0.1–0.3 nL of 0.5 mM alpha-Bungarotoxin (Tocris, Bristol, UK) in the ventral axial musculature. Zebrafish larvae were imaged using a custom spinning disk microscope (3i, Intelligent Imaging Innovations, Denver, CO, USA) equipped with a set of water-immersion objectives (Zeiss 20X, 40X, NA = 1). Recordings were acquired using Slidebook software at 10 Hz with a 488 nm laser. Gain and binning were manually optimized to maximize signal to noise ratio. Z-projection stacks showed full pattern of expression using Fiji (Schindelin et al.,2012). Thin-walled, borosilicate glass capillaries (Sutter Instruments, Novato, CA, USA) were pulled and fire-polished from a Flaming/Brown pipette puller (Sutter Instruments, Novato) to generate water jet stimulation pipettes. Stimulation pipettes were filled with external solution, connected to a pneumatic microinjector with vacuum pressure (WPI, Sarasota, USA), and positioned next to the preparation using motorized micromanipulators under the microscope. Water jet stimulations were either manually induced, or timed online in pClamp8.2 (Axon instruments). Stimulations were elicited every 2–3 min to reduce habituation. Positions of cells along the D-V axis were computed using Fiji and Matlab (Mathworks, USA). Calcium signals were extracted online using custom MATLAB scripts (Kevin Fidelin, Wyart Laboratory, Paris). Regions of interest (ROIs) were manually designed and raw fluorescence signals time series were extracted as the mean fluorescence from individual ROIs at each time point of the recording. DF/F calcium traces were generated and aligned to water jet stimuli in Matlab.

### In vivo intracellular recordings

6dpf zebrafish larvae were decapitated and pinned to a Sylgard coated recording chamber (Sylgard 184, Dow Corning, Midland, MI, USA) through the notochord with electrolytically sharpened tungsten pins. The skin was removed and the specimen was bathed briefly in a 10% formamide solution and subsequently washed in bath recording solution to eliminate spontaneous muscle twitching. For paired recordings, the dura was exposed by suctioning away dorsal muscle fibers with a glass pipette. Typically 3–7 segments of dorsal muscle were removed. Recording electrodes were fashioned from capillary glass (1.5 mm O.D., 1.1 ID, WPI, Sarasota, FL, USA) with a horizontal puller (P1000, Sutter Instruments, Novato, CA). Electrode resistances were 8–14 MΩ for CaP motor neurons and 2–5 MΩ for fast skeletal muscle fibers. To patch motor neurons, positive pressure (65 mmHg) was applied to the recording electrode via a pneumatic transducer (Fluke Biomedical DPM1B, Everett, WA). Once the electrode was driven through the dura in order to approach the targeted motor neuron, the positive pressure was reduced to 35 mmHg. Fast skeletal muscle fibers were exposed and subsequently patched by first removing the superficial layer of slow muscle fibers with a glass suction pipette. Motor neurons were held at − 65 mV in current clamp mode and 2 msec current injections of ~ 400 pA were used to evoke action potentials. Muscle cells were held at − 50 mV in voltage clamp mode. External bath recording solution contained the following (in mM), 134 NaCl, 2.9 KCl, 2.1 CaCl2-H20, 1.2 MgCl2, 10 Glucose, 10 HEPES with pH adjusted to 7.4, and osmolarity to 290 mOsm. Motor neuron and muscle cell internal solution contained the following (in mM), 115 K-Gluconate, 15 KCl, 2 MgCl2, 0.5 EGTA, 4 Mg-ATP, 10 HEPES pH 7.2, 290 mOsm. All reagents were obtained from Sigma-Aldrich (St. Louis, MO, USA) unless otherwise noted. Patch electrodes contained 40 μM Alexa Fluor 488 (Life Technologies Ltd., Paisley, UK). Physiological recordings were made with an Axopatch 700B amplifier and digitized with a Digidata 1440A (Molecular Devices, Fremont, CA, USA). pClamp software (Molecular Devices, Fremont, CA, USA) was used to acquire electrophysiological data. Motor neuron recordings were acquired at a sampling rate of 50 kHz and postsynaptic currents were acquired at 100 kHz. Recordings were low pass filtered at 2.2 kHz. Series resistance was monitored for muscle cell recordings and was < 10 MΩ. Data were analyzed with Clampfit (Molecular Devices, Fremont, CA, USA), Igor Pro 6.34 (WaveMetrics, Lake Oswego, OR), and Excel 2010 (Microsoft, Redmond, WA, USA). Summary data are presented as average ± SEM.

### Electron microscopy

6dpf larvae were fixed in 2% glutaraldehyde and 2% paraformaldehyde in cacodylate buffer 0.1 M pH 7.4 to which 3 mM of CaCl2 was added for 2 h at RT. Samples were washed 3 times in cacodylate buffer 0.1 M pH 7.4 and then post-fixed with 1% osmium tetroxide in distilled water for 1 h at 4 °C. After an extensive wash (3 × 10 min) with distilled water they were incubated for 1 h in 5% uranyl acetate in water. They were then dehydrated in a graded series of ethanol solutions (2x5min each): 50, 70, 80, 90, and 100%. Final dehydration was performed twice in100% acetone for 20 min. Samples were then progressively infiltrated with an epoxy resin, Epon 812® (EMS, Souffelweyersheim, France): 1 night in 50%resin 50%acetone at 4 °C in an airtight container, 2x2h in pure fresh resin at room temperature. They were embedded in the bottom of capsules (Beems size 3, Oxford Instruments, Saclay, France) and the resin was polymerized at 60 °C for 48 h in a dry oven. Blocks were cut with an UC7 ultramicrotome (Leica, Leica Microsystemes SAS, Nanterre, France). Semi-thin sections (0.5 μm thick) were stained with 1% toluidine blue in 1% borax. Ultra-thin sections (70 nm thick) were recovered either on copper (conventional morphology) or nickel (immunoelectron microscopy) grids and contrasted Reynold’s lead citrate. Ultrathin sections were observed with a Hitachi HT7700 electron microscope (Elexience, Verrière-le-Buisson, France) operating at 70 kV. Images were taken with an AMT41B camera at low (× 53,000), medium (× 70,000), and high (× 110,000) magnification, the last of which was used for quantification, done with ImageJ.

### Quantitative RT-PCR

Total RNA was extracted from previously phenotyped 6dpf embryos using a standard TRIzol reagent protocol (ThermoFisher Scientific). cDNA was then synthesized using the retrotranscription SuperScript III First-Strand Synthesis system kit (ThermoFisher Scientific) with the random hexamer primers. The qRT-PCR mix was prepared in technical triplicates with SYBR Green Master Mix (ThermoFisher Scientific) and run on an ABI PRISM 7900HT Real-Time PCR System (ThermoFisher Scientific) using *ef1a* and *rpl13a* as reference genes [47]. The analysis was performed according to the deltaCT quantification method and presented as a relation to wild-type levels (fold-change) [48].

### List of q RT-PCR primers

- dctn1a_Fwd: TCGAAGCTGA TGATCCCGTG
- dctn1a_Rev: TCCTGAGGGA GTGTGTGTGA
- dctn1b-fwd: GCAAAGGAGG AGAAGAGAGG
- dctn1b-rev: TGGAGAAGGC GATGGAC
- P22P24_Fwd: CACAAATACA CATTCAACAG CAGGAC
- P22P24_Rev: AGAGTTTCAT CCCACTGTGA AAACTG
- P25_Fwd: CTGTCCTTCC CCCAGAGACA
- P25_Rev: TCTGGCTGAG AGGGAGGAAT
- p50_Fwd: CCTCCAACGA GCCTGATGTT
- p50_Rev: TAGCGCTGAC GTGTTTGTCT
- ndel1b_Fwd: TACACCTGTG GGGAAGACCA
- ndel1b_Rev: TCCTTGCTGC CTGATCCTTG
- pafah1b1a_Fwd: CTTGTGCACC CTGGAGGAAA
- pafah1b1a_Rev: GTACGGAGCA GTCTTGTGGA
- pafah1b1b_Fwd: TGACACTGGT TGGCCATGAT
- pafah1b1b_Rev: AGTGTTCATG GGCACTGAGG
- bdnf_Fwd: CTTGAGGTGG AAGGGGAAGC G
- bdnf_Rev: GTAACGGCGG CTCCAAAGGC
- actr1.1-1_Fwd: GGGTCGGGAG TTATCAAGGC
- actr1.1-1_Rev: CCGGTGCTCCTCTGCTTTAG
- kif14_Fwd: CTCCAGCACA CCTCATGGAG
- kif14_Rev: TCCCTGGAGC TGAAAGGTCT
- rpl13a__Fwd: TCTGGAGGACTG TAAGAGGTTGC
- rpl13a__Rev: AGACGCACAATC TTGAGAGCAG
- ef1a_Fwd: CTGGAGGCCAGC TCAAACAT
- ef1a_Rev: ATCAAGAAGAGT AGTACCGCTAGCATTAC

### Statistics

Data compilation and analysis were done using Excel (Microsoft, USA) and graph generation was done using GraphPad Prism version 6 for Windows (GraphPad Software, USA). Using GraphPad, Student’s t-test was used for normally distributed data and Mann-Whitney U test was used for non-normally distributed data, when comparing mutants and wild-types. For comparison of multiple groups, SigmaPlot 11.0 integrated with SigmaStat 3.1 was used and a one-way ANOVA on ranks was performed, followed by Dunn’s multiple comparison procedure (pairwise). The Z-test was used to compare population proportions. Significance, set at *p* ≤ 0,05 (*), *p* ≤ 0,01 (**), *p* ≤ 0,001 (***).

## Results

### Establishing a model for Dynactin1 depletion

Previous work has yielded *mikre oko* ^*m632*^ (*mok*^*m632*^) a transgenic line harboring a point mutation within the coding sequence for *dynactin1a*, a zebrafish ortholog for *DCTN1* [37]. The C to T transition at nucleotide 2395 creates a premature stop codon and severely reduced *dynactin1a* mRNA in *mok*^*m632−/−*^ embryos [39]. A subsequent absence of a detectable truncated peptide suggests the mutation is amorphic or strongly hypomorphic [49]. Homozygous mutants larvae do not survive past the second week of development, as has been described before [38]. As *Dynactin1* loss-of-function has proven to be embryonic lethal in other models [50, 51], this suggests that maternal contribution of Dynactin1a in the zebrafish model is sufficient to ensure early survival. Maternally-provided protein is depleted by 4dpf [52] and we observed a nearly 70% reduction in protein level in homozygous embryos at 2dpf (Additional file 1: Figure S1b, c). This level is comparable to what has been achieved by cell culture siRNA silencing of *DCTN1* in previous studies [53].

At early stages, *mok*^*m632−/−*^ embryos are indistinguishable from their wild-type siblings, and by 4 days post-fertilization (dpf) exhibit smaller eyes with protruding lenses, a morphological phenotype resulting from cell death in the photoreceptor layer, also rendering them blind [38]. No additional morphological phenotype was visible in 6dpf homozygous mutant larvae (Additional file 1: Figure S1a) and heterozygous carriers are adult-viable and morphologically indistinguishable from wild-type siblings.

### Dynactin1a depletion does not affect initial development of the CaP motor neurons, but leads to growth defects and abnormal innervation of fast-twitch muscle fibers

In order to determine if reduced levels of Dynactin1a had an effect on the development of motor neurons, led to their degeneration, or impaired their function, we focused our attention on the trunk and tail, more specifically, the CaP motor neurons of the spinal cord [54]. The CaPs innervate fatigable type II (fast-twitch) muscle fibers as early as 22–24 h post-fertilization (hpf), forming an early but functional NMJ by 2dpf [55].

**Fig. 1:**
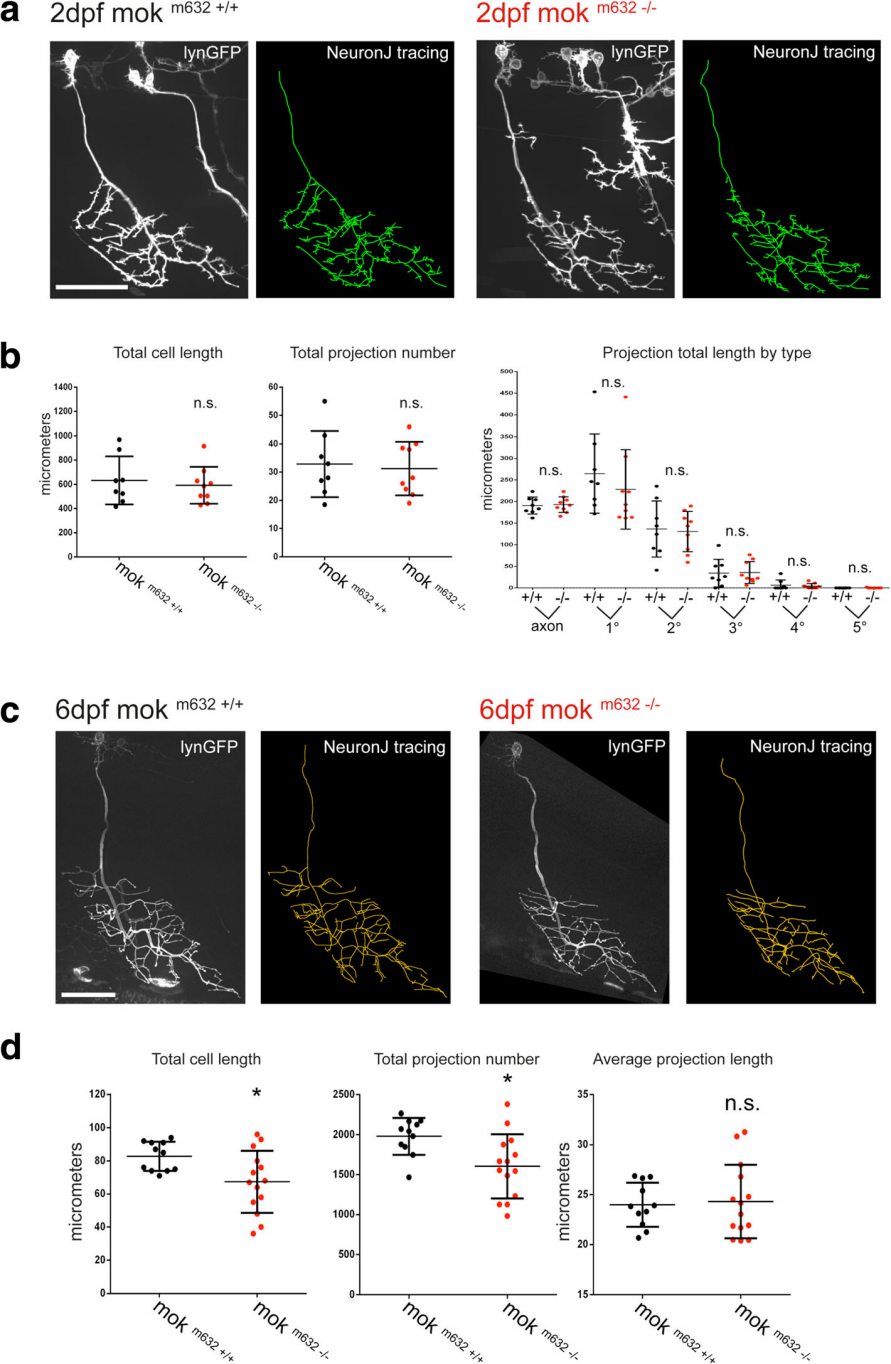
Dynactin1a depletion leads to proper initial development of the CaPs but impaired growth. Axonal morphology of CaP primary motor neurons in vivo at **a** 2dpf, and at **b** 6dpf visualized in confocal z-stack projections by single-cell expression of membrane-bound *mnx1:lyn-GFP,* with NeuronJ tracings of the axonal arbor. Quantification of the tracings for size (total cell length and projection number) and complexity (projection number by branch order) of CaPs, **c** showing no significant difference in size between cells of mutants and wild-type siblings at 2dpf, but **d** revealing that CaPs in 6dpf homozygous mutant larvae have a smaller arbor composed of fewer projections, which retain average length when compared with their wild-type siblings. All data presented as average +/− SD. (**b**: n cells wild-type, mutant = 9, 9; **d**: n cells = 11,14). Scale bar = 50 μm

We visualized CaP morphology in live embryos by single-cell expression of a membrane-bound fluorophore. This was achieved by injection in zygotes of a DNA construct where *lyn-GFP* was put under the control of the *mnx1* transcription factor, selectively expressed in postmitotic motor neurons of the spinal cord [56]. Comparing cell tracings in *mok*^*m632−/−*^ embryos with their wild-type siblings revealed that the complexity of axonal arbors, defined by total cell length, projection number, and complexity by branch order, was not altered at 2dpf (Fig. 1a, b). CaPs in 6dpf *mok*^*m632−/−*^ larvae exhibited a significantly smaller arbor, which was also less complex, as determined by a reduced total cell length and number of projections (Fig. 1c, d).

We next examined NMJ integrity by performing double-immunohistochemistry on fixed embryos. Presynaptic structure was revealed by labeling of Synaptotagmin-2 (Additional file 2: Figure S2a, c, in green) and the postsynaptic receptors were labeled by a fluorophore-conjugated α-bungarotoxin (Additional file 2: Figure S2a, c, in red), which binds irreversibly to the acetylcholine receptors (AChR) present on muscle fibers. Analysis revealed no change in colocalization or correlation of both signals in the 2dpf ventral root (Additional file 2: Figure S2b), indicating that pre- and postsynaptic components are well aligned and that CaPs, along with the other motor neurons present in the ventral root, properly innervated their target muscle in *mok*^*m632−/−*^ embryos at 2dpf. These results indicate that Dynactin1a depletion does not interfere with initial development of the CaPs, as the cell size and synaptic structure of the NMJ are conserved in 2dpf *mok*^*m632−/−*^ embryos. However, we found reduced coverage of pre- and postsynaptic markers in 6dpf *mok*^*m632−/−*^ larvae, consistent with the observed smaller arbor, but also reduced colocalization of both markers as shown by lower Pearson’s and overlap coefficients (Additional file 2: Figure S2d). As we did not detect orphan vesicles or receptors at 6dpf, our results suggest that the smaller arbor size is due to improper growth rather than degeneration.

These results indicate that depletion of Dynactin1a leads to proper migration and initial development of CaP motor neurons and their NMJ, but to growth defects at 6dpf associated with a compromised NMJ structural integrity.

### Dynactin1a depletion does not alter distribution of cargoes or axonal transport dynamics

Since Dynactin1 is thought to regulate and direct dynein activity, and acts as the dynactin complex’s only direct link with microtubules, we hypothesized that if dynein-mediated retrograde transport was reliant on Dynactin1a as an essential part of the dynactin complex, a depletion could reduce the number of assembled dynactin complexes available and cause defects in clearing of damaged organelles and detritus, or could affect signaling from the synapse. Alternatively, as Dynactin1 is known to be involved in the coordination of bidirectional movement [57], depletion could affect supply for a growing synapse by anterograde transport. We then sought to determine if Dynactin1a depletion caused axonal transport defects in CaP motor neurons of 2dpf embryos, and if this could be the cause of the reduced arbor size observed at 6dpf. To exclude the influence of an aberrant morphology on this process, we focused this analysis on 2dpf embryos, which still present normal CaP morphology,

We selected general cargo markers and generated fusion protein constructs to analyze the axonal transport dynamics of mitochondria (labeled by phb-GFP) [44], early endosomes (labeled by rab5c-GFP), late endosomes/multivesicular bodies (labeled by rab7-GFP), and recycling endosomes (labeled by rab11a-GFP) in vivo. To effectively target CaP motor neurons, we used the GAL4/UAS system and relied on injection of DNA constructs in the Tg(*mnx1:GAL4)* transgenic background with a co-expressed membrane-bound fluorophore reporter (UAS:tagRFP-Caax) to confirm cell type by morphology (Additional file 3: Figure S3a).

We first assessed cargo distribution in CaP cells, as disruption of retrograde transport could lead to the formation of aggregates or to abnormal distribution, where cargo would amass at one end of the distal end of the cell if only being transported anterogradely. Furthermore, transport defects could affect the fusion and fission of endosomal vesicles and mitochondria, a process that is essential to their function and if impaired, would alter their size. We quantified number, mean area, total area (coverage) of all labeled cargoes, as well as their axonal distribution with relation to the cell body in 2dpf CaPs (Additional file 3: Figure S3b). No significant difference was observed for these metrics in mok ^m632−/−^ embryos when compared with their wild-type siblings, and no aggregates were seen, indicating a normal distribution of cargoes.

In order to determine if Dynactin1a depletion modified axonal transport dynamics, we performed in vivo time-lapse imaging of single CaP axonal segments, located at mid-axon. This allowed the quantification of axonal transport of cargoes in single cells, by kymogram analysis of transport (Additional file 4: Figure S4a). We first classified each cargo trace into three net transport states based on their movement during the acquisition period, whether they were immobile (black), moving toward the cell body (retrograde, magenta) or moving toward the synapse (anterograde, cyan). We found no significant difference in the percentage of cargo in each state between mok ^m632−/−^ embryos and their wild-type siblings (Additional file 4: Figure S4b). We then quantified metrics such as area flux in the retrograde direction and in the anterograde direction (Additional file 4: Figure S4c, d), and axonal segment vesicle density (Additional file 4: Figure S4e). In addition, we also determined average run speed, length, and duration for runs in both the retrograde and the anterograde direction (example traces on Additional file 4: Figure S4a; Additional file 5: Figure S5). Surprisingly, we did not find any significant difference in these measurements when comparing mok ^m632−/−^ embryos with their wild-type siblings.

These results suggest that the extent of Dynactin1a depletion at 2dpf does not affect distribution, anterograde or retrograde axonal transport of mitochondria, and early, late, and recycling endosomes in CaP motor neurons.

### Cytoskeleton dynamics are not affected by Dynactin1a depletion

The growth defects observed at 6dpf could be due to the switch in growth signals between a migrating axon, directed by guidance cues [58, 59], and a maturing arbor, responding to local trophic signaling [60, 61]. Indeed, trophic signaling is essential for the growth and plasticity of CaP arbors. The actin cytoskeleton will form filopodia to search the environment for guidance cues, trophic signaling and adhesion molecules provided by the muscle fibers [62]. These structures get infiltrated by microtubules to form nascent branches, once stabilized by post-synaptic partners, [63], or are retracted when proper signal is absent [64]. Proteins localizing at microtubule +ends (like Dynactin1) are known to regulate the actin cytoskeleton and mediate the signaling of guidance cues during neuronal development [65].

**Fig. 2:**
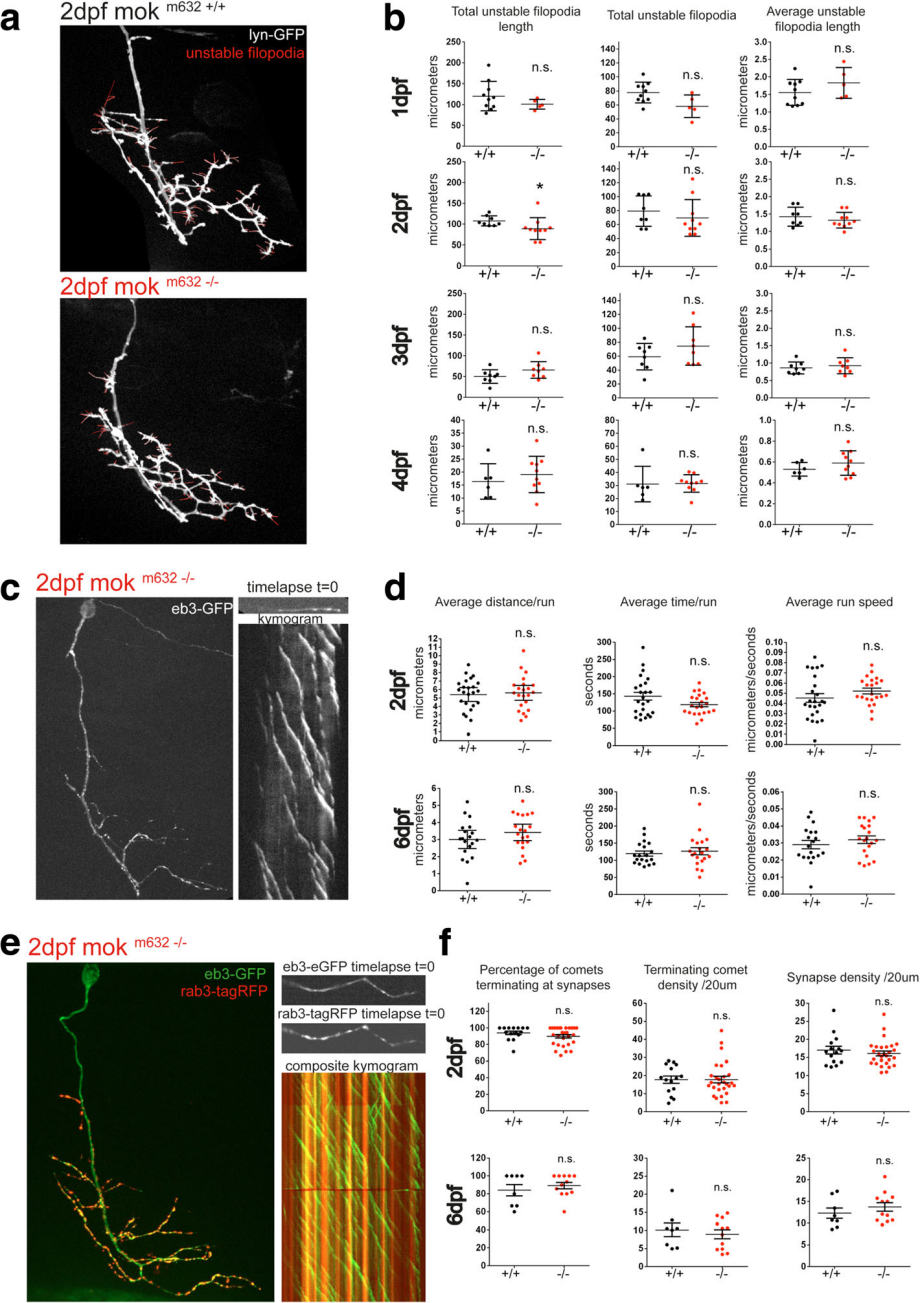
CaP growth defect is independent of cytoskeleton dynamics modulation. **a** Actin filopodia dynamics is assayed by time-lapse imaging of single CaP cell arbors expressing membrane-bound reporter lyn-GFP, from 2dpf to 4dpf. Example of confocal z-stack projection of a 2dpf CaP, with an overlay showing total unstable filopodia in red. **b** Quantification of filopodia dynamics over 10 min reveals no change in total unstable filopodia length, number or average length for 1dpf, 2dpf, 3dpf or 4dpf cells, with the exception of total unstable filopodia length at 2dpf, which was found to be slightly diminished. **c** Microtubule growth was determined by time-lapse imaging of eb3-GFP comets at both 2dpf and 6dpf. **d** Quantification of extracted kymograms shows no change in microtubule growth at either timepoint, as determined by average distance, duration and average speed of comet runs. **e** Microtubule capture at putative synapses was assayed by expression of a synaptic marker (rab3-tagRFP, in red) simultaneously with eb3-GFP (in green) at 2dpf and 6dpf. **f** Quantification of microtubule capture at putative synapses, density of terminating eb3 comets or putative synapses per axonal segment reveal this process was not affected by loss of Dynactin1a. Data presented as average +/− SEM. (**b**: 1dpf *n* = 10,5; 2dpf *n* = 8,10, 3dpf *n* = 8,8, 4dpf *n* = 6,10; **d**: 2dpf *n* = 24, 22, 6dpf *n* = 22,20; **f**: 2dpf *n* = 15, 28, 6dpf *n* = 8,12)

In order to determine if Dynactin1a depletion interfered with the actin cytoskeleton stabilization or with trophic signaling, we monitored filopodia dynamics via in vivo time-lapse imaging of CaP cells expressing lynGFP (Fig. 2a). We quantified the amount of unstable filopodial extensions by means of total length, total number and average length of unstable filopodia at 1dpf, 2dpf, 3dpf and 4dpf, but found no significant differences between mok ^m632−/−^ embryos and their wild-type siblings aside from a slight reduction in total length of unstable filopodia at 2dpf (Fig. 2b). To confirm that loss of Dynactin1a did not alter trophic signaling, we also performed axonal transport analysis of vesicles containing the survival/suicide low-affinity trophic receptor p75 (*ngfra*), and found no difference in ratios, area flux or transport metrics (Additional file 6: Figure S6). Because we only observed a slight reduction in total length of unstable filopodia at 2dpf, without additional defects in other metrics like average length and filopodia number, as well as no in changes trophic signaling, we deemed this change not biologically relevant and moved on to the analysis of the microtubule cytoskeleton.

As Dynactin1 is known to bind microtubules via its CAP-Gly domain, and to act at as an anti-catastrophe factor at plus-tips (+tips) [66], we then investigated microtubule growth by quantification of EB3 comets. This +tip protein binds the labile end of the tubules during bouts of assembly and the resulting runs, or “comets” were visualized in vivo by time-lapse imaging of fusion proteins in single CaP arbors at 2dpf and 6dpf (Fig. 2c). Quantification of comet metrics like average distance, time and speed of runs, did not reveal any changes in either timepoints when comparing mok ^m632−/−^ embryos with their wild-type siblings (Fig. 2d), suggesting microtubule growth and stability are not affected by Dynactin1a depletion.

Microtubule capture at synapses is known to rely on the dynein/dynactin complex and its interaction with adhesion molecules to anchor microtubules at the membrane and promote synapse stability [67]. We therefore co-expressed our EB3 comet construct described previously with rab3-tagRFP, a marker for putative synapses [45], to label both growing microtubules and putative synapses within the same CaP arbor in live 2dpf and 6dpf embryos (Fig. 2e). We then performed time-lapse imaging to determine the density of terminating comets and of synapses in terminal branches of the axonal arbor. The ratio of microtubule capture, defined by comets terminating their run at putative synapse sites, for mok ^m632−/−^ embryos was unchanged when compared with their wild-type siblings (Fig. 2f). This suggests that synaptic microtubule capture, while dependent on interaction between dynein and the dynactin complex [67], is not affected by Dynactin1a depletion.

These results indicate that the growth defects observed in 6dpf CaP motor neurons upon Dynactin1a depletion do not result from impaired modulation of the actin or microtubule cytoskeleton, and that the cell has both the potential for sensing and the support for trophic signaling in mok ^m632−/−^ embryos.

### Dynactin1a depletion leads to synapse instability at the NMJ

Synapses are known to be necessary for the stabilization of new branches in a growing axonal arbor [67] and previous studies suggested a role for Dynactin1a in synapse growth and stability in *Drosophila* [68, 69]. While we did not observe a change in microtubule capture, defects in local organization of the presynaptic structure could lead to instability and impair growth of the CaPs.

**Fig. 3:**
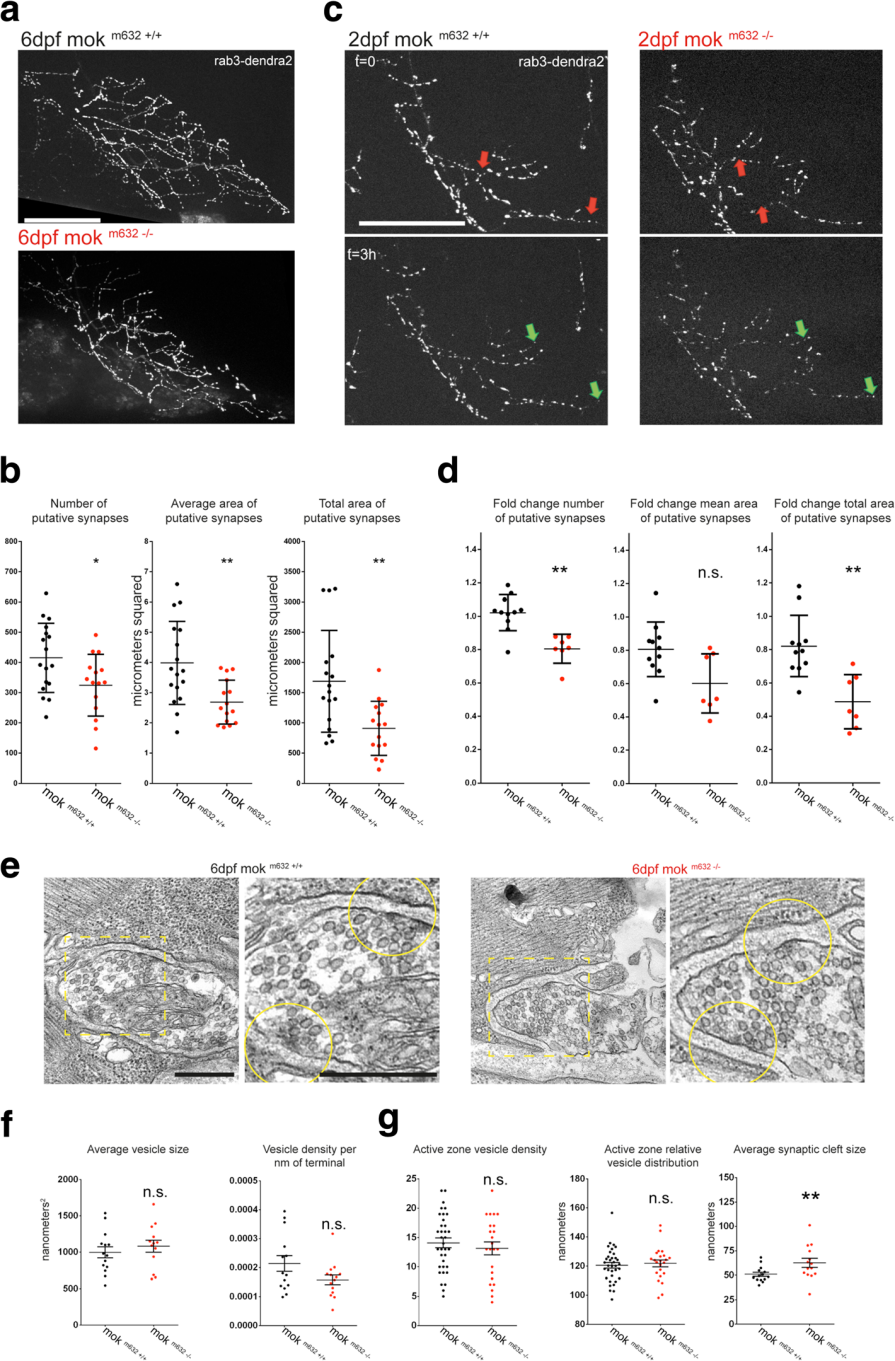
Dynactin1a depletion leads to synapse instability at 2dpf, reduced synaptic density at 6dpf and ultrastructural changes. **a** Putative synapses are visualized with rab3-dendra2 labeling in single CaP cells at 6dpf. **b** Synaptic coverage in the arbors, determined by number, average area and total area, of putative synapses is reduced in 6dpf larvae in homozygous mutant embryos when compared with their wild-type siblings. **c** Synapse stability at 2dpf was assayed by imaging cell arbors over a period of 3 h, where comparison of the initial stack (t = 0) with the subsequent one (t = 3 h) for the same cell was used to determine the number of stable synapses. Examples of synapses added and lost are indicated in green and red arrows respectively. **d** Quantification is presented as fold-change and reduced in homozygous mutant embryos for number and total area, but not for mean area of putative synapses, when compared with their wild-type siblings. **e** Electron micrograph of a transverse section of the 6dpf NMJ, with close-up (dashed yellow box), showing active zones (center of yellow circle) in NMJ synapses of mok ^m632−/−^ larvae and their wild-type siblings. **f** No changes were detected in number of synaptic vesicles and average vesicle size when measured in the synaptic terminal. **g** Normal density and distribution of vesicles was also observed around the active zones (yellow circle perimeter), however the synaptic clefts were significantly wider at mok ^m632−/−^ larvae active zones. Data shown as **b**) **d**) average +/− SD, **f**) **g**) average +/− SEM. (**c**: n cells = 11,7; **d**: n cells = 17,15, **f**: n slices = 14, 14; **g**: n active zones = 34,22). Scale bar **a**) **c**) 50 μm; **e**) 500 nm

Double immunohistochemistry on whole-mount preparations revealed overall conserved NMJ structural integrity for all motor neurons present in the ventral root at 2dpf (Additional file 2: Figure S2 a, b). In order to observe CaP synapses specifically, we labeled single neurons by injecting *pUAS-dendra2-rab3* in the Tg(*mnx1:GAL4)* transgenic background. We observed the size and coverage of putative synapses in single cells of live mok ^m632−/−^ and wild-type embryos at 2dpf (Additional file 7: Figure S7a), where no difference was found in number, average size, and arbor coverage (total area) of putative synapses between mok ^m632−/−^ embryos and their wild-type siblings (Additional file 7: Figure S7b). However, labeling of CaP synapses in live 6dpf larvae (Fig. 3a) revealed that the smaller cell arbors seen in mok ^m632−/−^ embryos contain less synapses, which are also of smaller size (Fig. 3b).

We then performed time-lapse imaging on CaP arbors over a period of 3 h, a benchmark lifetime for putative synapse stabilization [67] (Fig. 3c) in order to assess if Dynactin1a depletion led to instability at 2dpf which could explain the reduced number of NMJ synapses in 6dpf arbors. While we had not found significant differences for number of synapses between wild-type and mok ^m632−/−^ embryos at 2dpf (as shown in Additional file 7: Figure S7b), comparison of the same cell between the two timepoints (initially and 3 h later) allowed quantification of synapses lost and gained over this period, represented as fold-change. We found reduction in number and arbor coverage (total area) of putative synapses in 2dpf mok ^m632−/−^ embryos when compared with their wild-type siblings, while average size (mean area) was maintained (Fig. 3d). In addition to microtubule capture, which was found to be unchanged in our mutant (Fig. 2e, f), synapse stability also relies on interaction with adhesion molecules. We investigated localization of N-Cadherin at NMJ synapses by using previously described BAC transgenic *Tg(cdh2:Cdh2-GFP)* [41]. At the NMJ, N-Cad-GFP forms a puncta at the center of the presynaptic structure and this localization was not perturbed in 2dpf (Additional file 8: Figure S8a) or 6dpf mok ^m632−/−^ embryos (Additional file 8: Figure S8b). As N-Cadherin is known to be involved in synapse stabilization by mediating cell-cell interactions, our results suggest that synapse instability is not due to impaired localization of this adhesion molecule at the synapse, although defects could still arise from compromised interactions upon depletion of Dynactin1a.

These results indicate that Dynactin1a depletion impairs synapse stability at 2dpf, independently of proper localization of N-Cadherin, and leads to impaired stabilization of nascent branches of growing CaP motor neuron arbors and a reduced number of putative synapses at 6dpf.

### Ultrastructural analysis of NMJ synapses support a local role for Dynactin1a

Visualization of the 6dpf NMJ by electron microscopy (Fig. 3e) confirmed the lack of aggregates in synaptic terminals of mok ^m632−/−^ larvae which could have supported defects in axonal transport. In addition, we found that the synaptic vesicles were of similar average size and density throughout the terminal in both mutant and wild-type larvae (Fig. 3f). Although they appeared less clustered, it was not possible to determine if organization of vesicle pools was maintained in mok ^m632−/−^ larvae. When looking at the active zone (AZ) perimeter (yellow circle Fig. 3e), we observed normal density of synaptic vesicles, which exhibited a similar distribution in relation to the center of the AZ (Fig. 3g), suggesting no change in the availability of vesicles as part of the readily-releasable pool. However, the synaptic cleft as measured between the neuron membrane and the muscle at the level of the AZs, was significantly wider in mok ^m632−/−^ larvae (Fig. 3g). These observations indicate that despite the lack of changes in microtubule capture at synapses or in the localization of N-cadherin, Dynactin1a could have a role in active zone structure and organization.

### Synapse instability leads to impaired NMJ function and locomotion defects

**Fig. 4:**
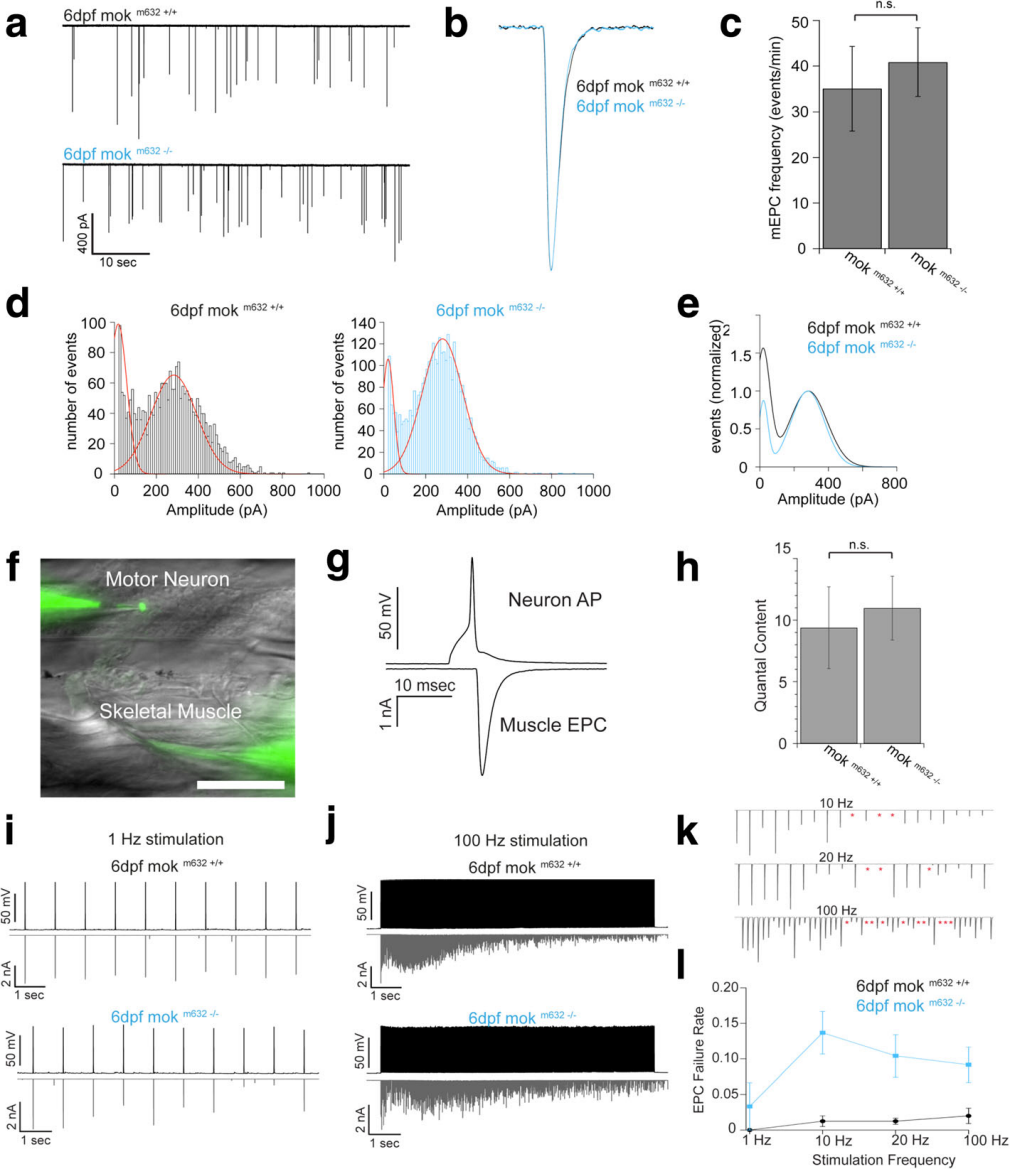
NMJ function is impaired by severely reduced levels of Dynactin1a. **a** Sample traces of spontaneous miniature end plate currents (mEPCs) from fast-twitch muscle fibers. **b** Normalized individual mEPCs from mok ^m632−/−^ larvae (blue trace) and their wild-type siblings (black trace). **c** Average mEPC frequency recorded from wild-type and mutant mok ^m632−/−^ larvae. **d** mEPC amplitude histogram for wild-type and mok ^m632−/−^ larvae with Gaussian functions (red traces). **e** Normalized Gaussian fits from wild-type (black trace) and mok ^m632−/−^ larvae (blue trace). **f** Paired motor neuron skeletal muscle fiber recording each patched and filled with AlexaFluor 488 (calibration bar = 100um). **g** Motor neuron action potential is evoked by a 2 msec current injection (upper trace) and subsequent muscle EPC is recorded (lower trace). **h** Average quantal content (evoked EPC amplitude/mEPC amplitude for wild-type and mok ^m632−/−^ larvae. Sample traces from paired neuron – muscle whole cell recordings for wild-type and mok ^m632−/−^ larvae with stimulus frequencies of **i** 1 Hz and **j** 100 Hz. **k** Magnification of evoked EPCs from mok ^m632−/−^ larvae recordings demonstrating postsynaptic EPC failures occurring at 10, 20 and 100 Hz (red asterisks). **l** Evoked EPC failure rate is significantly higher in mok ^m632−/−^ (blue trace) than in wild-type larvae (black trace) for paired recordings 10 Hz, 20 Hz and 100 Hz. Data shown as mean +/−SEM. (**c**: average = 35.04 ± 9.25, *n* = 2638 events from 15 fish/ average = 40.86 ± 7.53, *n* = 4903 events from 24 fish; **d**: *n* = 2638 events from 15 fish/ *n* = 4903 events from 24 fish; **d**: WT peak 1 average = 18.35, WT peak 2 average = 283.19, mutant peak 1 average = 20.42, mutant peak 2 average = 280.00; **h**: QC = 9.39 ± 3.31, *n* = 8 pairs/ QC = 10.98 ± 2.59, *n* = 18 pairs; **l**: *n* = 8 pairs/ *n* = 9 pairs)

To determine if synapse instability and wider synaptic clefts had functional consequences on synaptic transmission, we performed whole-cell voltage clamp recordings of individual fast-twitch skeletal muscle fibers and monitored spontaneous miniature end plate currents (mEPCs, Fig. 4a). We observed that mEPCs from the muscle of 6dpf mok ^m632−/−^ larvae and of their wild-type siblings shared similar kinetics (Fig. 4b, Additional file 9: Figure S9a, b), frequencies (Fig. 4c) and amplitudes (Fig. 4d) of mEPC, as well as a similar quantal size for spontaneously released single vesicles from the CaP [70] (Fig. 4e), suggesting that even with severely reduced levels of Dynactin1a, the synaptic vesicles in CaPs motor neurons are released normally, that they contain usual quantities of neurotransmitter and that the postsynaptic ACh receptors on fast-twitch muscle fibers are not affected.

In order to investigate the consequence of Dynactin1a depletion on evoked neurotransmission, we next performed paired whole-cell patch clamp recordings of CaP motor neurons and of their target fast-twitch skeletal muscle [70] (Fig. 4f). In current clamp mode, a short pulse of current (2 msec, ~400pA) was injected into the CaP motor neuron to elicit an action potential (AP) (Fig. 4g, upper trace) and the subsequent EPC was recorded in an innervated muscle fiber in voltage clamp mode (Fig. 4g, lower trace). While quantal content for the AP-evoked EPCs in muscle fibers were not different in mok ^m632−/−^ larvae (Fig. 4h), we observed variability in EPC amplitude (Fig. 4i, j) and half of the motor neurons tested displayed a higher number of EPC failures. These failures, where CaPs fail to release neurotransmitters, occur at a higher rate during 10 Hz, 20 Hz and 100 Hz stimulation (traces shown for 1 Hz in Fig. 4i and 100 Hz in Fig. 4j, Fig. 4k, labeled failures in mutant traces, Fig. 4l quantification) (additional frequencies Additional file 9: Figure S9c).

**Fig. 5:**
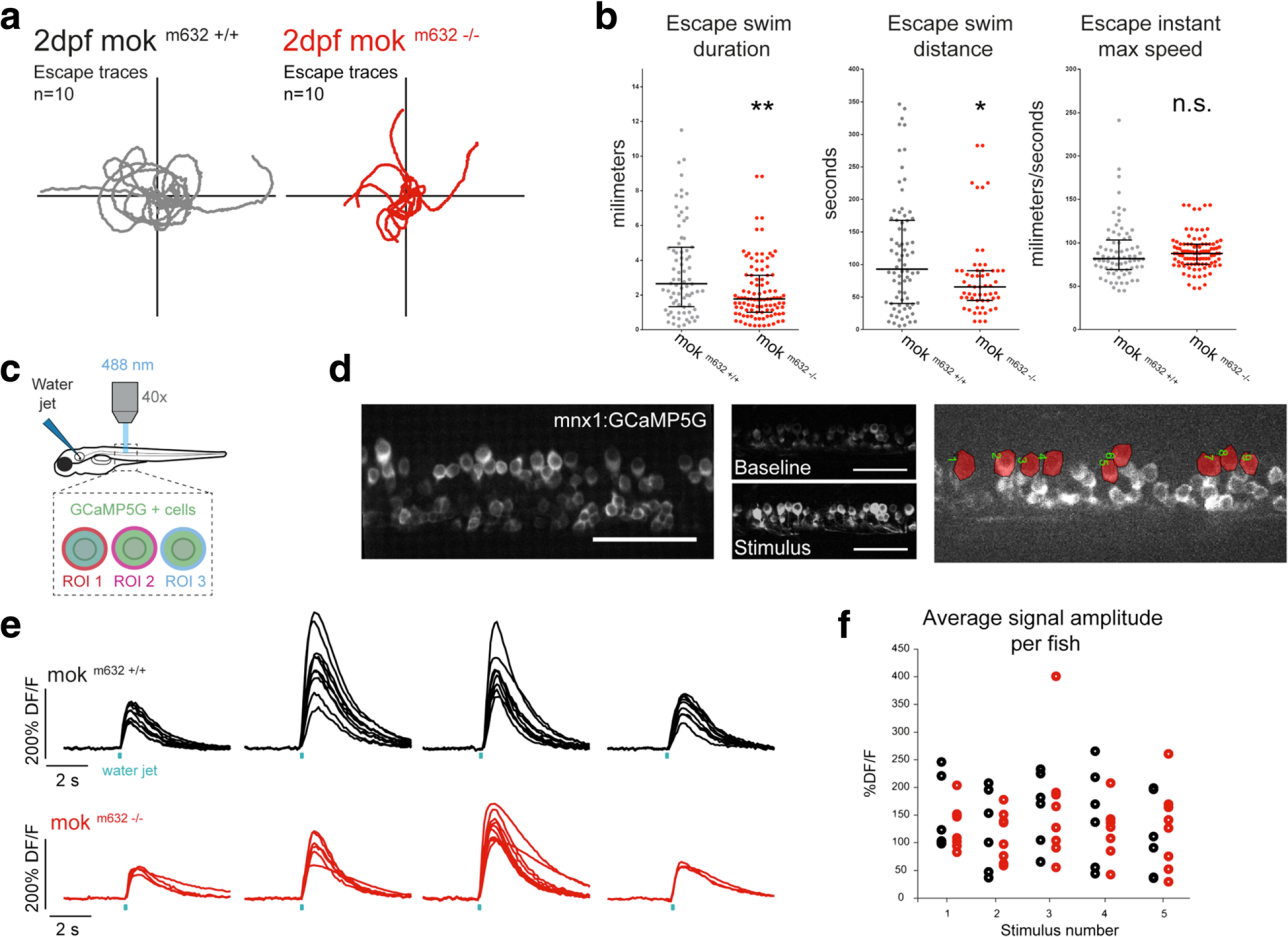
NMJ dysfunction leads to behavioral deficits. **a** NMJ functional defects lead to impaired locomotor behavior in 2dpf embryos as determined by touch-evoked escape response assay. Escape traces extracted from video tracking of escape swimming episodes following the presentation of a stimulus for 10 embryos per genotype shown here as an example. **b** Quantification of escapes reveal that Dynactin1a depletion leads to impaired locomotion determined by reduced escape duration and distance, but without altering maximum instant speed. **c** Calcium imaging of fictive escape responses in motor neurons expressing GCaMP5 was performed in the spinal cord upon presentation of a water jet stimulus. **d** GCaMP5 expression was confined to motor neurons and analysis of calcium signals was performed on dorsally-located primary motor neurons (region of interest in red). **e** Example of calcium signals obtained from primary motor neurons including CaP motor neurons in mok m632^−/−^ larvae (red) and their wild-type siblings (black) at 4dpf; one trace per cell, four fictive escape responses are represented to show response variability. **f** Maximum DF/F amplitude signal in dorsal motor neurons averaged per fish and plotted according to the stimulation number, showing proper recruitment of spinal cord motor neurons despite reduced levels of Dynactin1a. Data shown as b) median +/− interquartile range (**b**: *n* = 76,101; **f**: n embryos/n cells = 6/63, 8/44) Scale bar 100 μm

Because we could not perform paired recordings at 2dpf, we then tested if the observed synaptic instability led to locomotion defects at 2dpf. We performed a touch-evoked escape response (TEER) assay where embryos produce stereotypical swimming episodes in response to touch stimuli (traces of escapes Fig. 5a). The escapes produced by mok ^m632−/−^ embryos were of shorter duration and covered less distance than the ones of their wild-type siblings (Fig. 5b). Furthermore, the maximum instant speed, a readout for muscle function [71], was not affected by Dynactin1a depletion (Fig. 5b).

As swimming is a complex behavior that requires synchronous activity of spinal cord neurons, we used optogenetics to exclude the possibility that the observed phenotype arose from impaired circuit connectivity upstream of the CaPs. We probed recruitment of motor neurons in the spinal cord during fictive swimming upon presentation of water jet stimuli (Fig. 5c) while monitoring neuronal activity in the spinal cord with a genetically-encoded calcium indicator (*Tg(mnx1:GAL4; UAS:GCaMP5,* Fig. 5d). No differences in maximum DF/F amplitude signal for each cell analyzed were noted in 4dpf mok ^m632−/−^ larvae when compared with their wild type siblings (Fig. 5e). This indicates that CaPs have normal calcium transient when stimulated, and that there are no connectivity defects upstream of the CaP motor neurons.

These results suggest that Dynactin1a depletion leads to electrophysiological abnormalities at the NMJ, where the synaptic instability observed in 2dpf CaPs of normal morphology leads to functional deficits at the NMJ culminating in abnormal locomotion, without affecting muscle function, and where 6dpf mok ^m632−/−^ larvae displayed varying EPC amplitudes and a higher rate of failures in response to action potential, while still maintaining normal spontaneous release kinetics and quantal content.

### Expression of human Dynactin1-eGFP rescues defects in a cell-autonomous manner

**Fig. 6:**
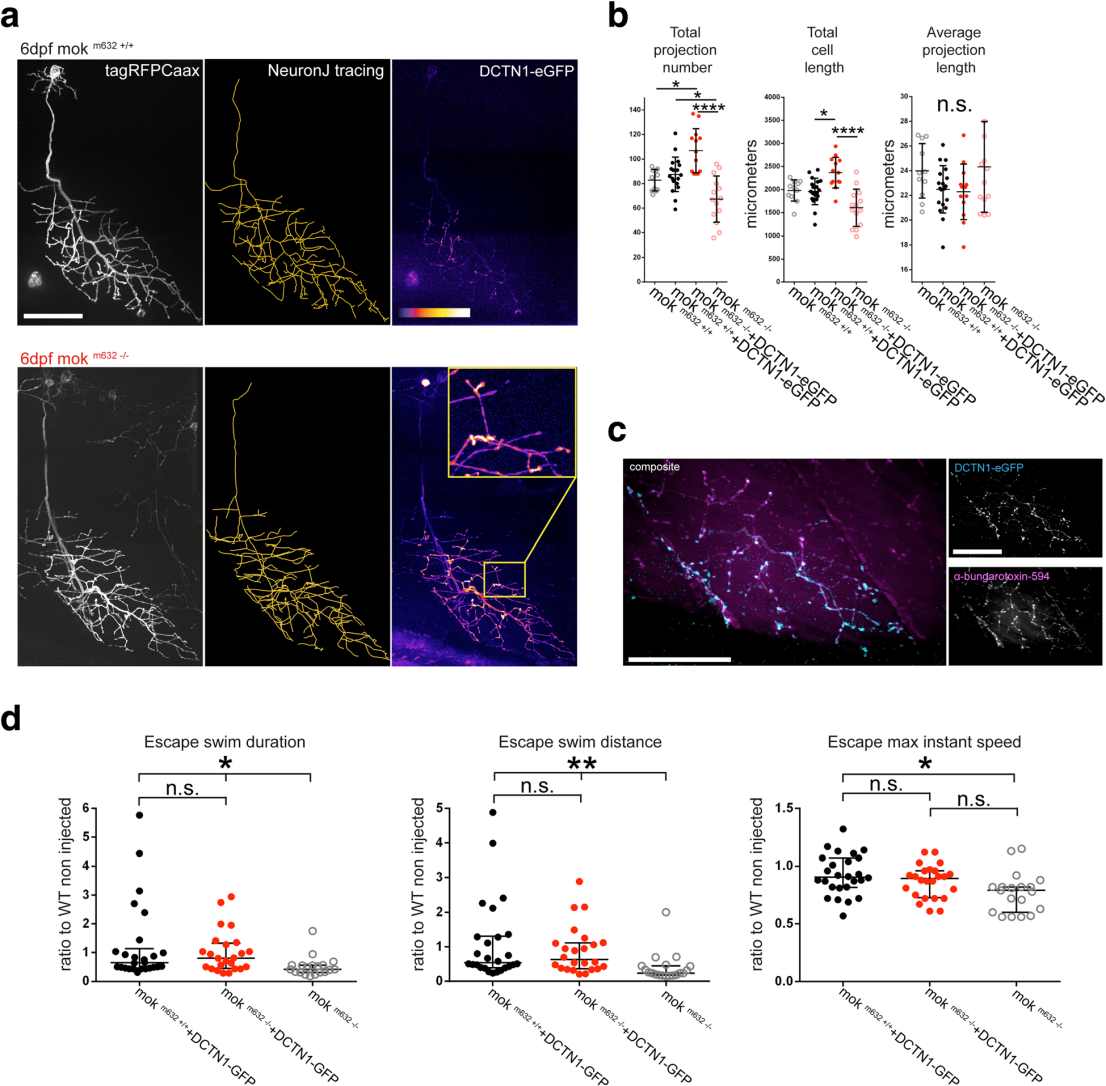
Overexpression of human wild-type DCTN1 rescues the CaP growth defects at 6dpf and the behavioral deficits at 2dpf. **a** CaP morphological defects seen at 6dpf in mutant larvae are rescued by single-cell overexpression of DCTN1-GFP. **b** Quantification of cell tracings show recovery of arbors size in rescued mutant larvae, as determined by total projection number and total cell length, without affecting average projection length. Overgrowth is due to lack of competition by neighboring mutant cells. **c** DCTN1-GFP is found to accumulate at synaptic sites (close-up of heatmap), **d** as confirmed by colocalization (in white) in double immunohistochemistry of DCTN1-GFP (anti-GFP in cyan) with post-synaptic ACh receptors (α-bungarotoxin, in magenta). **d** Overexpression of exogenous DCTN1-GFP by injection of 400 ng/ul RNA rescued the NMJ functional defects leading to impaired locomotor behavior in 2dpf embryos. Touch-evoked escape response was quantified and is shown as ratios relative to the average values obtained for wild-type escapes for duration, distance, and maximum instant speed. Data shown as c) average +/− SD, d) median +/− interquartile range (**b**: n cells = 19, 13; **d**:n embryos = 26, 24, 19). Scale bar = 50 μm

To confirm that the phenotype observed in our mok ^m632−/−^ larvae did not involve the muscle fiber or surrounding glia, we specifically overexpressed wild-type human Dynactin1-eGFP (DCTN1-eGFP) together with an E2A-linked membrane-bound fluorophore reporter (tagRFP-Caax) in single CaP neurons. At 2dpf, overexpression of DCTN1-eGFP did not have an effect on CaP morphology either in mok ^m632−/−^ embryos or their wild-type siblings (Additional file 10: Figure S10 a, b). At 6dpf however, the human protein was able to rescue the morphological phenotype in mok ^m632−/−^ larvae (Fig. 6a), as mutant CaPs had larger, more complex axonal arbors than wild-type CaPs, based on total cell length and number of projections, while maintaining average projection length (Fig. 6b). This overgrowth in rescued CaPs is most likely due to lack of competition from neighboring cells, lacking Dynactin1-eGFP, and still exhibiting a reduced axonal arbor due to Dynactin1a depletion. Overexpression of Dynactin1-eGFP in 6dpf wild-type CaPs did not affect cell morphology, similar to what was observed at 2dpf. These results suggest that loss of Dynactin1a is acting in a cell-autonomous manner to cause a morphological phenotype and that human wild-type Dynactin1-eGFP can rescue these defects in mok ^m632−/−^ embryos. Furthermore, we observed an enrichment of Dynactin-eGFP at synaptic termini at 6dpf arguing for a local role at this site (Fig. 6a, c).

### Expression of human Dynactin1-GFP rescues behavioral deficits

To confirm that expression of human wild-type Dynactin1 rescued neuromuscular function in mok^m632−/−^ embryos, we synthesized RNA encoding Dynactin1-GFP and injected it in 1–2 cell stage eggs. This resulted in a broad expression of the protein throughout the embryo, as detected by GFP signal (Additional file 10: Figure S10c), allowing us to perform the 2dpf TEER assay described previously. The escapes produced by mok ^m632−/−^ and mok ^m632−/+^ embryos injected with Dynactin1-GFP were of similar duration and distance while being significantly different from uninjected mok ^m632−/−^ (Fig. 6d). These results show that exogenous Dynactin1-GFP expression can rescue the locomotion abnormalities described in our mutants and thus rescue neuromuscular function.

Overall, our results in CaP motor neurons of the zebrafish spinal cord support a role for Dynactin1a in NMJ dysfunction, where it acts locally to ensure synapse stability and function, independently of its role in regulating dynein activity, directing axonal transport or in modulating cytoskeleton dynamics.

## Discussion

In this study, we characterized a progressive Dynactin1a depletion in vivo*,* focusing specifically on its effect on primary motor neurons of the zebrafish spinal cord. We report here that loss of Dynactin1a is sufficient to impair primary motor neuron function, where CaP motor neurons exhibit impaired growth of axonal arbors, neuromuscular junction synapse instability and functional abnormalities culminating in locomotion defects.

A striking result of this study is the lack of detectable impact on axonal transport and cytoskeleton dynamics in *dynactin1a* mutants. It is now well demonstrated that genetic mutations can lead to upregulation of related genes and potentially to functional compensation for loss of the protein encoded by the mutated gene [72].

Due to genome duplication, the zebrafish has another paralog for *DCTN1*: *dynactin1b* (ZV11; ENSDARG00000056753) that could account for such a compensation. Previous assemblies of the zebrafish genome (ZV8 and ZV9) predict a shorter protein produced from this gene, homologous to the p135 isoform of Dynactin1 [73] lacking the microtubule-binding CAP-Gly domain as a result of alternative splicing. As we did not observe a change in the expression of this paralog in *mok*^*m632−/−*^ larvae by qRT-PCR (Additional file 1: Figure S1d), we conclude that loss of *dynactin1a* does not trigger a genetic compensation by *dynactin1b,* however it still could be acting in functional redundancy. We were unable to amplify by RT-PCR a dynactin1b cDNA containing the predicted CAP-GLY domain (exons 1–3) in either *mok*^*m632−/−*^ or wild-type embryos (data not shown), further supporting the hypothesis that the domain is not included in the wild-type mRNA, and does not get spliced in following *dynactin1a* silencing. Consequently, *dynactin1b* likely leads to a protein similar to the 135 kDa isoform, which could explain why we do not see drastic changes in axonal transport. Indeed, both isoforms are found in neuronal populations [74] and bind dynein in independent complexes [73]. Because dynein alone binds well to stable, detyrosinated microtubules, while it requires both the dynactin complex (with the full-sized Dynactin1) and adaptor BicD2 to interact with tyrosinated microtubules such as the ones found at the dynamic (+) ends [75], the absence of full-sized Dynactin1a in presence of the short p135 equivalent Dynactin1b would likely only affect the initiation and not the processivity of transport [76].

We also did not detect changes in modulation of the cytoskeleton dynamics in mok ^m632−/−^ embryos, another well-described function of Dynactin1 within the dynactin complex. Indeed, our mutants were found to efficiently stabilize microtubules at + ends, necessary for the growth of an axonal arbor, and to capture them at new synapses, a function which rely on interaction of the dynactin complex with the dynein motor and NCAM-180 [66, 67]. Following the initial migration of the growth cone to reach its target muscle, the cell relies on cues from the environment in the form of trophic factors acting in a feedback loop with NMJ activity. BDNF is known to act as a retrograde signal to stimulate the maturation of the NMJ synapses by promoting arbor outgrowth and branching and increasing the production of synaptic vesicle proteins [77]. We probed actin filopodia dynamics to determine if the cell was unable to locally detect trophic signaling. These metrics were also found to be unaffected by Dynactin1a depletion, as was the axonal transport of the survival/suicide trophic receptor p75 (*ngfra*). Furthermore, we did not find altered levels of *bdnf* mRNA (Additional file 1: Figure S1d)*,* which could have indicated compensation for the growth defects by the muscle target. This suggests that Dynactin1a depletion does not lead to changes in the capacity of the cell to detect or respond to trophic signaling and that our phenotype is independent of modulation of cytoskeleton dynamics.

Although transport and cytoskeleton dynamics appeared unaffected, we detected synapse instability and locomotion abnormalities at 2dpf, before the apparition of morphological defects, suggesting impaired NMJ function. Synapses are known to be necessary for the stabilization of nascent branches of a growing arbor [67]. As CaPs in mok ^m632−/−^ larvae have a reduced arbor size, containing a lower number of putative synapses which were of smaller size. Thus, a higher loss of synapses due to instability not compensated by new synapse formation can explain the observed growth defects and the reduction in putative synapse number at 6dpf. While changes in axonal transport or cytoskeleton dynamics would have easily explained the observed phenotype because of the necessity of retrograde transport and cytoskeleton modulation for signaling and degradation, our evidence suggests this is not the mechanism at play upon Dynactin1a depletion.

The locomotion defects detected in 2dpf mok ^m632−/−^ embryos by behavioral assay (TEER) indicate that the synapse instability observed at this early stage is sufficient to induce functional deficits at the NMJ. Injection of RNA encoding Dynactin1-GFP to obtain a broad but transient overexpression led to the rescue of this behavioral phenotype. It is important to note that the observed locomotion deficits, while statistically significant, were less severe than what has been described in previous ALS models [78–80]. In patients, impairment of NMJ function is reported to arise before the onset of motor neuron degeneration and clinical symptoms in early ALS [81]. This is consistent with our observations as Dynactin1a depletion leads to synapse instability and slight locomotion deficits at 2dpf, before the apparition of a morphological phenotype in CaP motor neurons and loss of NMJ structural integrity at 6dpf.

Electrophysiological recordings provided a closer look at NMJ synaptic dysfunction, showing that the release machinery is functional at the 2dpf and 6dpf mok ^m632−/−^ synapses and that the quantity of neurotransmitter contained in individual vesicles was not altered. In addition, no changes were found in the release kinetics or receptor properties. However, paired-recordings of the CaP-fast-twitch muscle fiber at 6dpf revealed that mok ^m632−/−^ NMJs have variable amplitudes and a higher rate of response failure to action potentials, when stimulated at 10, 20 and 100 Hz, similar to what has been described for the *FUS* loss-of-function ALS model [78].

Ultrastructural analysis of the NMJ synapses in 6dpf mok ^m632−/^ larvae confirmed that we did not have distal accumulation of aggregates, which would be expected in the event of impaired retrograde axonal transport, and showed that the mutant synaptic terminals contained vesicles which were of similar density and average size as the one of their wild-type siblings. We also observed that the active zone perimeter contained similar numbers of available vesicles (readily-releasable), however it was not possible to determine if the formation of synaptic vesicle pools, namely the reserve and recycling pools, was conserved. The synaptic cleft, composed of domains connecting the pre- and postsynaptic side of the synapse, was however wider in mok ^m632−/^ larvae at the level of the active zones, suggesting that active zone organization might be disturbed following Dynactin1a depletion. Although we did not investigate this mechanism in more detail, this Dynactin1 could be involved in generating force to maintain membrane apposition by interacting with adhesion molecules or by helping recruit or maintain functional pools in synaptic termini.

In our synapse stability assay, we used Rab3, a protein associated with vesicles at active zones, to label putative synapses. This protein is essential for neurotransmission, with a known role in calcium-evoked exocytosis, and interaction with the SNARE complex [82], and a role in synaptic vesicle formation and trafficking [83]. Moreover, impaired active zone scaffolding could lead to ineffective coupling and recruitment of synaptic vesicles, or could affect calcium channel clustering, explaining the failures in response during repeated action potentials seen in our paired-recordings. Indeed, evoked release (action-potential-mediated) relies on a nanodomain active zone organization for efficient neurotransmission, whereas spontaneous release, which was unaffected in our mutant, can occur at varied areas of the terminal [84]. Hence we speculate that the synapse instability and EPC amplitude variability, as well as the higher failure rate reported here is due to improper formation of active zones following depletion of Dynactin1a, either by interaction with adhesion molecules or by recruitment or maintenance of functional vesicle pools.

Along with the reduced *DCNT1* mRNA and protein levels reported in sporadic ALS patients [26], missense mutations in *DCTN1* have been reported in ALS patients [28–30, 32]. In the context of a dominant inheritance, these missense mutations could likely lead to haploinsufficiency, as one out of three outcomes of missense mutation [85]. In further support of this hypothesis, it was reported that animal models for Dynactin1 mutations lead to a reduction in protein expression, for instance in the G59S mice [50], or the G38S flies [51], however the effect of other ALS-related mutations on protein expression was not studied.

Because many of the ALS-linked mutations were also found in controls [28, 30, 32], and due to their rarity and the variability of clinical presentation of ALS patients, causality was not established, however it was suggested that these mutations could act as risk factors and compound other rare variants in an oligogenic etiology of ALS [31]. Indeed, DCTN1 expression was found to be downregulated in sporadic ALS patient postmortem spinal cord tissue as an early event preceding degeneration as it was observed in neuronal populations that were well preserved and without pathological markers for degeneration [25]. Our zebrafish model exhibited an initial reduced expression of Dynactin1, followed by a gradual depletion over the course of a few days, thus representing depletion kinetics of interest in the context of investigating the role of this protein in ALS pathogenesis.

## Conclusions

The in vivo characterization of the morphogenesis and function of motor neurons in zebrafish embryos and larvae depleted for Dynactin1a point toward a local role for this protein in stabilizing the neuromuscular synapses, impairing its function, without leading to motor neuron death. This role appears to be independent of Dynactin1’s known functions associated with the dynein motor in axonal transport or cytoskeleton dynamics modulation, possibly due to the likely presence of the shorter p135 isoform. Because our probing of interactions with cytoskeletal components or adhesion molecules did not reveal anomalies, a candidate approach of possible synaptic interactors of Dynactin1a would be necessary to help further understand the mechanism at play leading to NMJ dysfunction in this model. The defects reported here are milder than what has been described for established zebrafish ALS models investigating pathogenic mutations, but represent targeted impairments which are consistent with early disease presentation. We therefore propose that Dynactin1a depletion represents an early event in NMJ degeneration and that ALS-related mutations in this gene are likely not causative but indeed have a place in the oligogenic etiology of ALS pathogenesis.

## Additional files

Additional file 1: **Figure S1.** mok ^m632−/−^ embryo morphology at 6dpf, Dynactin1 protein quantification at 2dpf and qRT-PCR expression in mok ^m632−/−^ larvae. a) Wild-type sibling and homozygous mutant embryo morphology at 6dpf; close-up showing a dorsal view of the head to emphasize previously described eye phenotype. b) Western blot of maternally-contributed Dynactin1 in 2dpf mok ^m632−/−^ embryo (detected with anti-DCTN1 antibody from Origene, TA346929), c) quantified against gamma-tubulin at 32% wild-type level. d) Quantification of 3 biological replicates of qRT-PCR levels from 6dpf mok ^m632−/−^ larvae mRNA relative to the average wild-type levels obtained for 6dpf ^m632+/+^ larvae mRNA (presented as fold change) shows no compensation by *dynctin1b* or *kif14*, no change in the expression of other subunits of the dynactin complex (p22/24, *p25, p50*, *actr1*), no change in other known regulators of the dynein motor complex (*ndel1b, pafah1b1a/1b1b)*, and no changes indicative of trophic compensation (*bdnf)*. (TIF 45716 kb)

Additional file 2: **Figure S2.** mok ^m632−/−^ embryo and larvae NMJ structural integrity. a) Double immunohistochemistry reveals the integrity of the NMJ at 2dpf by coverage and colocalization of presynaptic structures (anti-synaptotagmin2, in green) and postsynaptic Ach receptors (α-bungarotoxin, in red). b) Quantification of the colocalization shows normal NMJ structure of the ventral root at 2dpf by both Pearson’s coefficient and the overlap coefficient. c) NMJ structure is also affected at 6dpf, with d) reduced coverage in pre- and postsynaptic components, as well as reduced colocalization. All data presented as average +/− SD; (b: n embryos = 12, 14; d: n larvae = 11,19) (TIF 32575 kb)

Additional file 3: **Figure S3.** Dynactin1a depletion does not alter cargo distribution. a) Example of cargo labeling, here for late endosomes (rab7-GFP, in green) co-expressed with a membrane-bound reporter (tagRFP-Caax, in red) in a single CaP motor neuron. b) The quantification of cargo size and coverage is done by cell compartment (axon, arbor), and distribution from the cell body is analyzed in the axon for mitochondria (phb-GFP), early endosomes (rab5c-GFP), late endosomes/multivesicular bodies (rab7-GFP) and recycling endosomes (rab11a-GFP). No difference is found for vesicle or organelle number, mean area, total area or distribution between homozygous mutants and their wild-type siblings at 2dpf. Data shown as average +/− SD. (n cells b: mitochondria *n* = 7,6, rab5c *n* = 5,7; rab7 *n* = 7,9; rab11a *n* = 10,9). (TIF 19764 kb)

Additional file 4: **Figure S4.** Dynactin1a depletion does not alter axonal transport dynamics. a) Example of in vivo timelapse imaging still (t = 0) and extracted kymogram with labelled runs (anterograde cyan, retrograde magenta). b) Kymogram analysis of cargo states reveals no change in dynamics (immobile in black, anterograde in cyan and retrograde in magenta) at 2dpf. In addition, no change was observed in retrograde or anterograde area flux c) and d), or for cargo density e) except for mitochondria which was slightly increased. Data shown as average +/− SEM. (*n* = kymogram c, d, e: mitochondria *n* = 5,17, rab5c *n* = 7,7; rab7 *n* = 11,14; rab11a *n* = 7,11). (TIF 24821 kb)

Additional file 5: **Figure S5.** Dynactin1a depletion does not alter transport run dynamics. Additional transport metrics for all cargo shows no change in average run speed, length or duration of retrograde and anterograde runs for a) mitochondria, b) rab5c vesicles, c) rab7 vesicles, or d) rab11 vesicles. Data shown as average +/− SEM. (*n* = kymogram a: *n* = 5,17; b: *n* = 7,7; c: *n* = 11,14; d: *n* = 7,11). (TIF 26989 kb)

Additional file 6: **Figure S6.** Transport of p75 receptor (*ngfra*) is not affected by loss of Dynactin1a. In vivo timelapse imaging of vesicles tagged with ngfra-eGFP fusion protein show no changes in a) transport states, b) area flux and vesicle density, c) average run speed, length and duration of retrograde and anterograde runs of the trophic receptor in 2dpf CaP motor neurons. All data presented as average +/−SEM. (n kymogram = 10,9). (TIF 16813 kb)

Additional file 7: **Figure S7.** Synapse distribution and size at 2dpf is not affected by loss of Dynactin1a. a) Putative synapses are visualized with rab3-dendra2 labeling in single CaP cells at 2dpf. b) Distribution is not affected, as determined by number, average area and total area, of putative synapses. All data shown as average +/− SD (b: n cells = 26,20). (TIF 12772 kb)

Additional file 8: **Figure S8.** N-Cadherin localization is not altered at mok^m632−/−^ adherens junctions of the NMJ. a) Confocal projection of live 2dpf Tg(cdh2:Cdh2-GFP; mokm632; mnx1:Gal4) embryos showing N-Cadherin-GFP located at the center of a CaP NMJ synapse, determined by presence of post-synaptic AChR labelled with conjugated α-bungarotoxin (bath application, in red). b) Whole-mount immunohistochemistry of 6dpf Tg(cdh2:Cdh2-GFP; mokm632; mnx1:Gal4) larvae showing N-Cadherin-GFP (anti-GFP, in green) located at the center of an NMJ synapse, which was co-labeled to show pre-and postsynaptic structures (respectively, synaptotagmin2 in red and conjugated α-bungarotoxin in blue). Boxes show close-up of the synaptic structures, surrounding the N-Cadherin puncta. (TIF 40868 kb)

Additional file 9: **Figure S9.** Loss of Dynactin1a leads to abnormal physiological properties of the NMJ. Additional metrics of NMJ whole-cell recordings showing no change in a) Tau decay and b) rise time of 6dpf mEPC recordings in mok^m632−/−^ larvae. c) Additional frequencies for paired-recordings of CaP-fast twitch muscle fibers at 6dpf showing similar failure rates of mutant NMJs. (TIF 18204 kb)

Additional file 10: **Figure S10.** Overexpression of human wild-type DCTN1 does not affect initial development of CaP motor neurons and exogenous expression is confirmed by increase in GFP signal. a) Overexpression of human wild-type DCTN1-GFP (heatmap), along with a membrane-bound reporter (tagRFP-Caax, traced in green), is obtained in a cell-autonomous manner and does not accumulate at synapses. b) Overexpression does not affect initial growth of CaP motor neurons in either mutant embryos or their wild-type siblings at 2dpf, as determined by total projection number, total cell length and average projection length. c) DCTN1-GFP RNA expression is confirmed by GFP detection in 2dpf embryos by fluorescent microscopy in the 488 nm channel, image shown here for non-injected embryos with red bar for histogram generation. Quantification confirms higher fluorescent signal in injected (400 ng/ul DCTN1-GFP) vs non injected wild-type embryos. All data shown as average +/− SD (b: *n* = 8,14; c: *n* = 10,5) (TIF 27508 kb)

## Data Availability

The data generated during this study is available upon request.

## Abbreviations

AChR: Acetylcholine receptors
ALS: Amyotrophic lateral sclerosis
AZ: Active zone
CaP: Caudal primary
DCTN1: Dynactin subunit 1
dpf: Days post-fertilization
hpf: Hours post-fertilization
mEPCs: Miniature end plate currents
*mok*^*m632*^: *mikre oko* ^*m632*^
NMJ: Neuromuscular junction
TEER: Touch-evoked escape response
