# Supplementary figures and images for: Dynactin1 depletion leads to neuromuscular synapse instability and functional abnormalities
Source: Mol Neurodegener. 2019 Jul 10;14:27. doi: 10.1186/s13024-019-0327-3 (PMC6617949; doi:10.1186/s13024-019-0327-3)

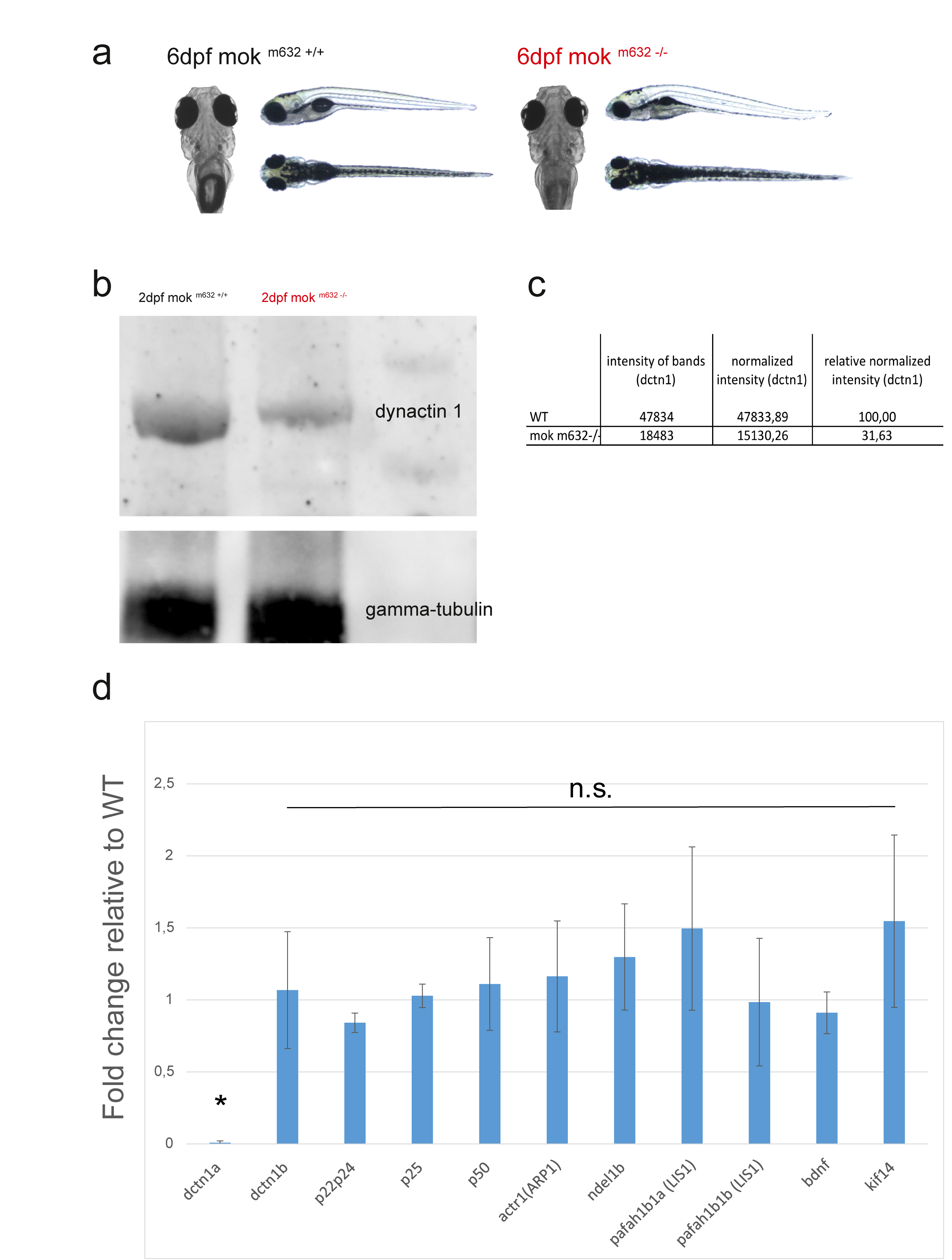

Supplement: Supplementary file 1 — Figure S1. mok m632−/− embryo morphology at 6dpf, Dynactin1 protein quantification at 2dpf and qRT-PCR expression in mok m632−/− larvae. a) Wild-type sibling and homozygous mutant embryo morphology at 6dpf; close-up showing a dorsal view of the head to emphasize previously described eye phenotype. b) Western blot of maternally-contributed Dynactin1 in 2dpf mok m632−/− embryo (detected with anti-DCTN1 antibody from Origene, TA346929), c) quantified against gamma-tubulin at 32% wild-type level. d) Quantification of 3 biological replicates of qRT-PCR levels from 6dpf mok m632−/− larvae mRNA relative to the average wild-type levels obtained for 6dpf m632+/+ larvae mRNA (presented as fold change) shows no compensation by dynctin1b or kif14, no change in the expression of other subunits of the dynactin complex (p22/24, p25, p50, actr1), no change in other known regulators of the dynein motor complex (ndel1b, pafah1b1a/1b1b), and no changes indicative of trophic compensation (bdnf). (TIF 45716 kb) [file 13024_2019_327_MOESM1_ESM.tif]

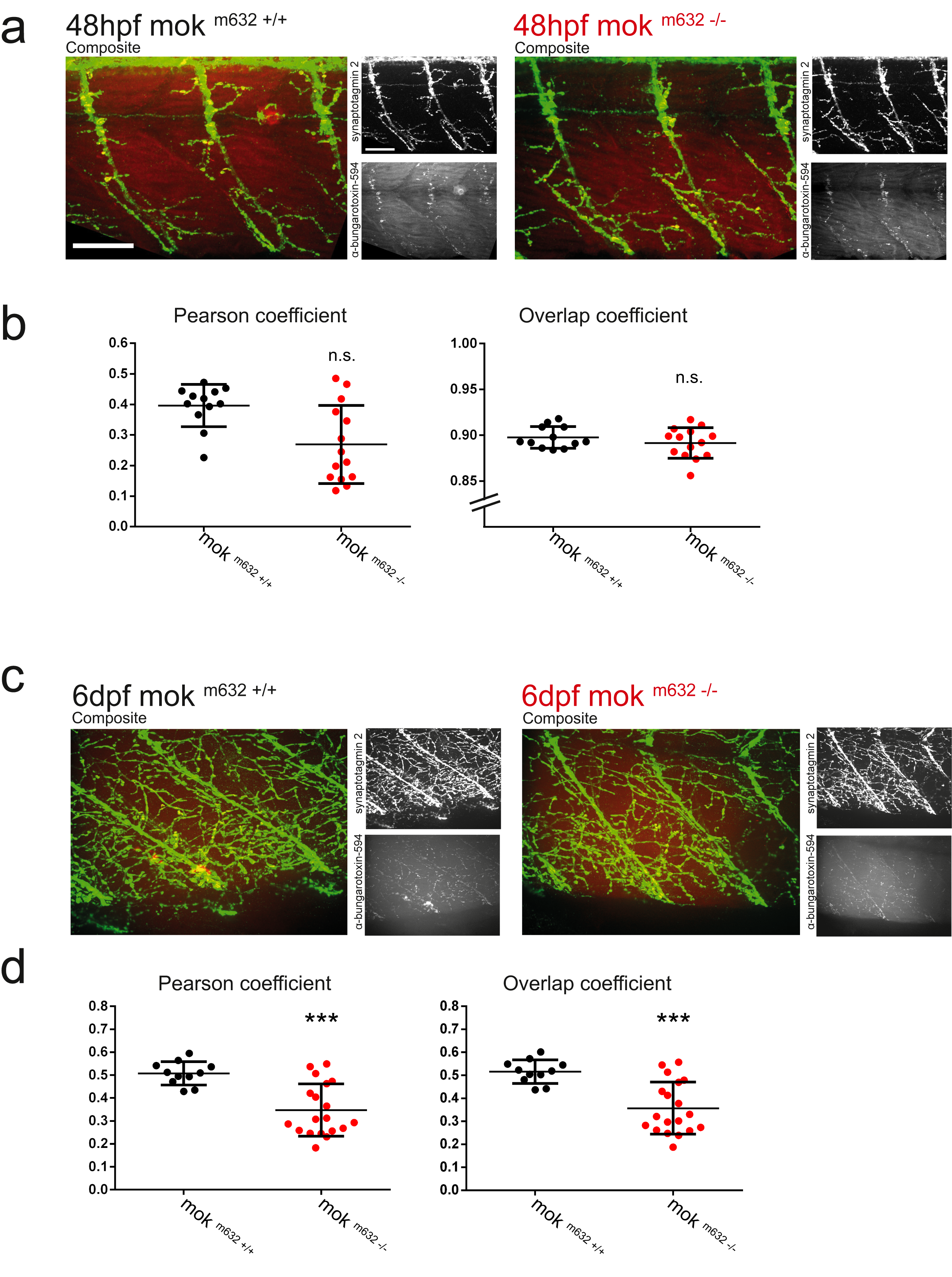

Supplement: Supplementary file 2 — Figure S2. mok m632−/− embryo and larvae NMJ structural integrity. a) Double immunohistochemistry reveals the integrity of the NMJ at 2dpf by coverage and colocalization of presynaptic structures (anti-synaptotagmin2, in green) and postsynaptic Ach receptors (α-bungarotoxin, in red). b) Quantification of the colocalization shows normal NMJ structure of the ventral root at 2dpf by both Pearson’s coefficient and the overlap coefficient. c) NMJ structure is also affected at 6dpf, with d) reduced coverage in pre- and postsynaptic components, as well as reduced colocalization. All data presented as average +/− SD; (b: n embryos = 12, 14; d: n larvae = 11,19) (TIF 32575 kb) [file 13024_2019_327_MOESM2_ESM.tif]

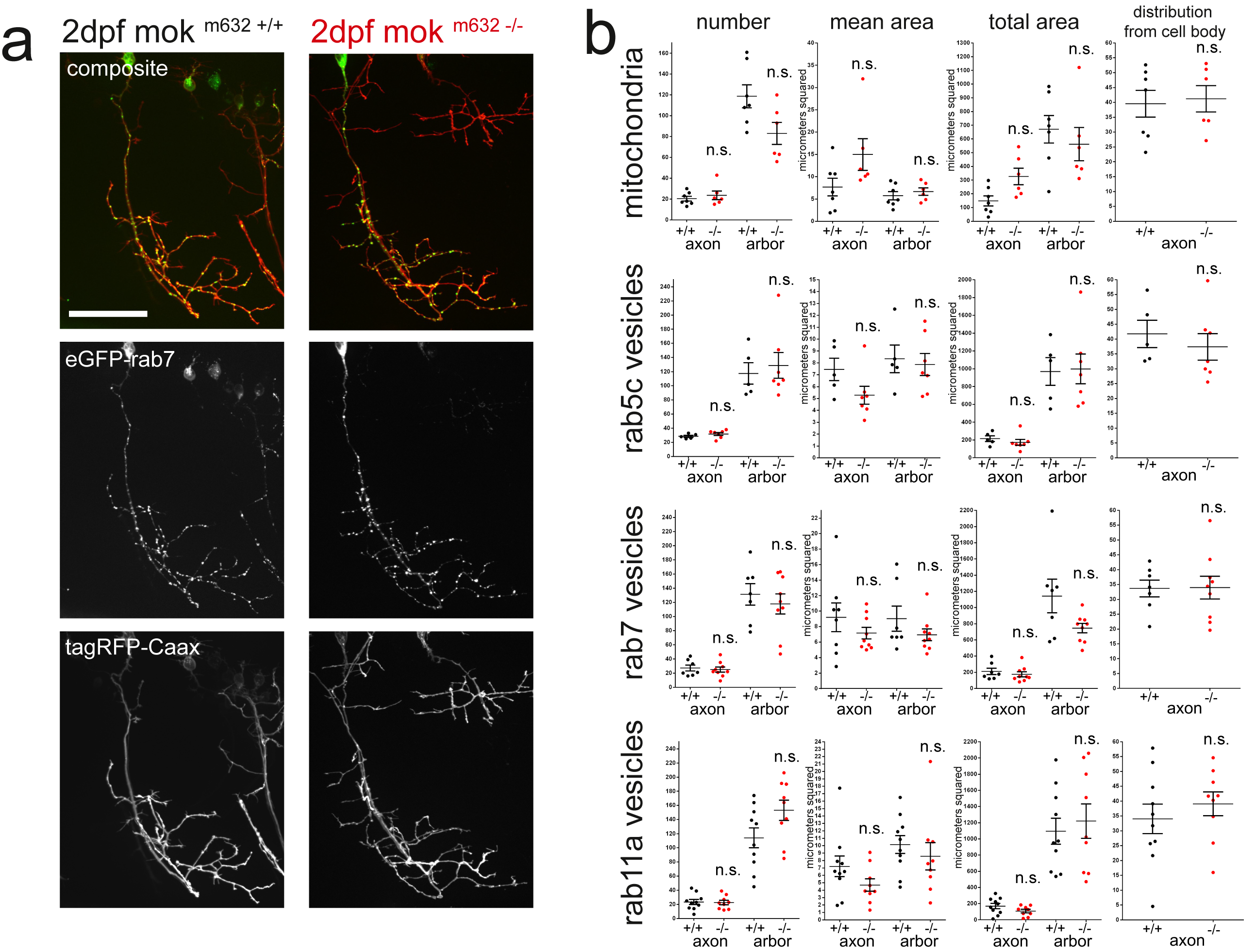

Supplement: Supplementary file 3 — Figure S3. Dynactin1a depletion does not alter cargo distribution. a) Example of cargo labeling, here for late endosomes (rab7-GFP, in green) co-expressed with a membrane-bound reporter (tagRFP-Caax, in red) in a single CaP motor neuron. b) The quantification of cargo size and coverage is done by cell compartment (axon, arbor), and distribution from the cell body is analyzed in the axon for mitochondria (phb-GFP), early endosomes (rab5c-GFP), late endosomes/multivesicular bodies (rab7-GFP) and recycling endosomes (rab11a-GFP). No difference is found for vesicle or organelle number, mean area, total area or distribution between homozygous mutants and their wild-type siblings at 2dpf. Data shown as average +/− SD. (n cells b: mitochondria n = 7,6, rab5c n = 5,7; rab7 n = 7,9; rab11a n = 10,9). (TIF 19764 kb) [file 13024_2019_327_MOESM3_ESM.tif]

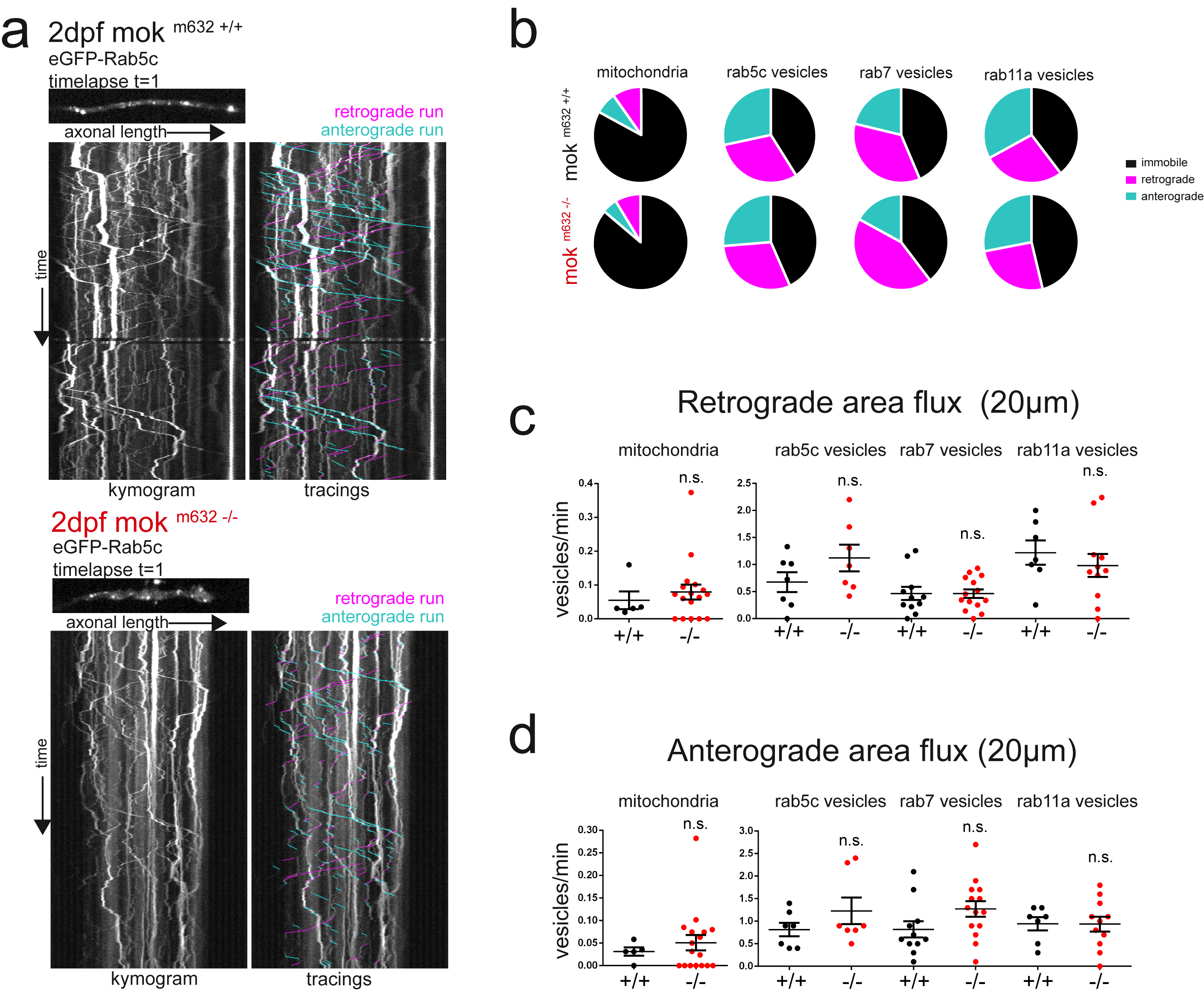

Supplement: Supplementary file 4 — Figure S4. Dynactin1a depletion does not alter axonal transport dynamics. a) Example of in vivo timelapse imaging still (t = 0) and extracted kymogram with labelled runs (anterograde cyan, retrograde magenta). b) Kymogram analysis of cargo states reveals no change in dynamics (immobile in black, anterograde in cyan and retrograde in magenta) at 2dpf. In addition, no change was observed in retrograde or anterograde area flux c) and d), or for cargo density e) except for mitochondria which was slightly increased. Data shown as average +/− SEM. (n = kymogram c, d, e: mitochondria n = 5,17, rab5c n = 7,7; rab7 n = 11,14; rab11a n = 7,11). (TIF 24821 kb) [file 13024_2019_327_MOESM4_ESM.tif]

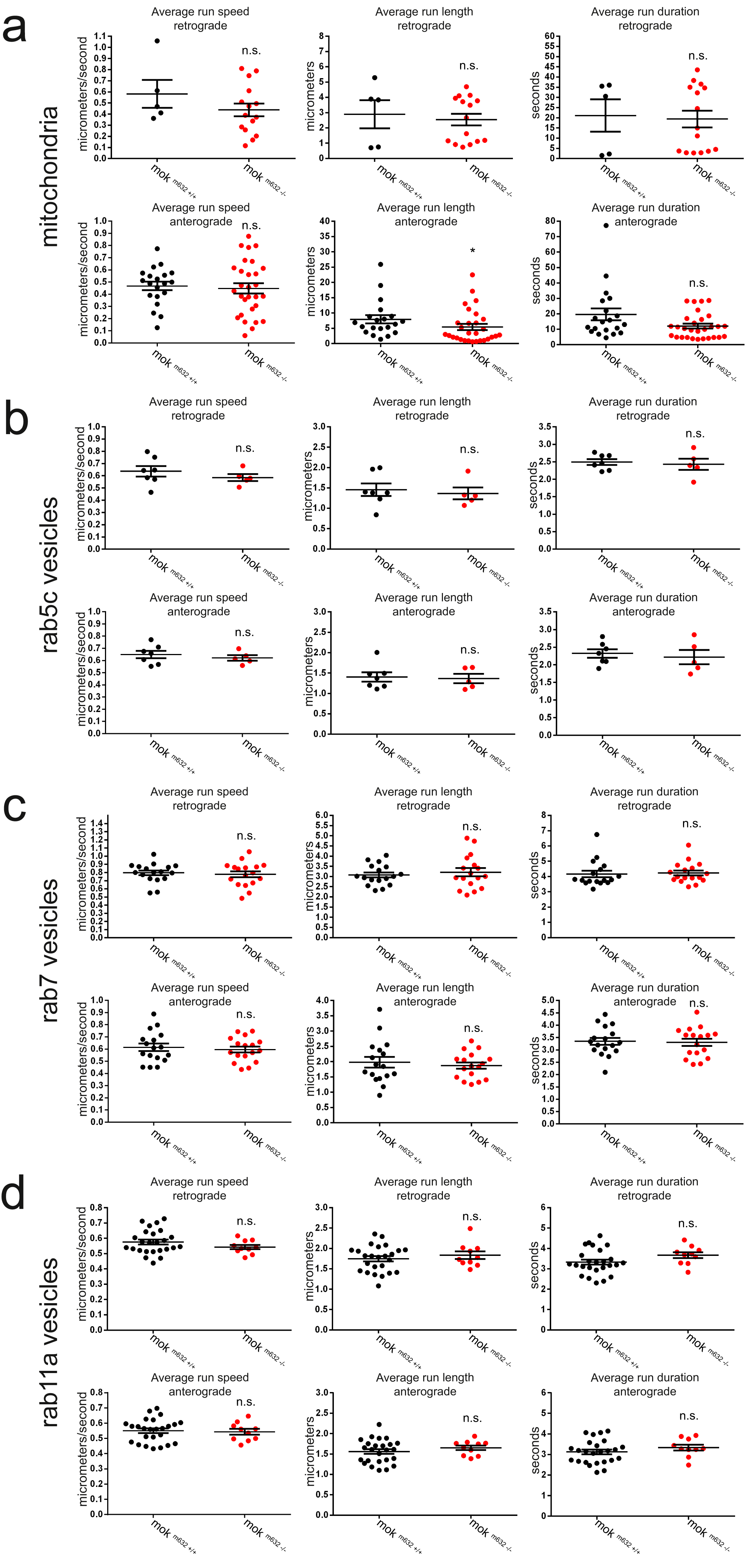

Supplement: Supplementary file 5 — Figure S5. Dynactin1a depletion does not alter transport run dynamics. Additional transport metrics for all cargo shows no change in average run speed, length or duration of retrograde and anterograde runs for a) mitochondria, b) rab5c vesicles, c) rab7 vesicles, or d) rab11 vesicles. Data shown as average +/− SEM. (n = kymogram a: n = 5,17; b: n = 7,7; c: n = 11,14; d: n = 7,11). (TIF 26989 kb) [file 13024_2019_327_MOESM5_ESM.tif]

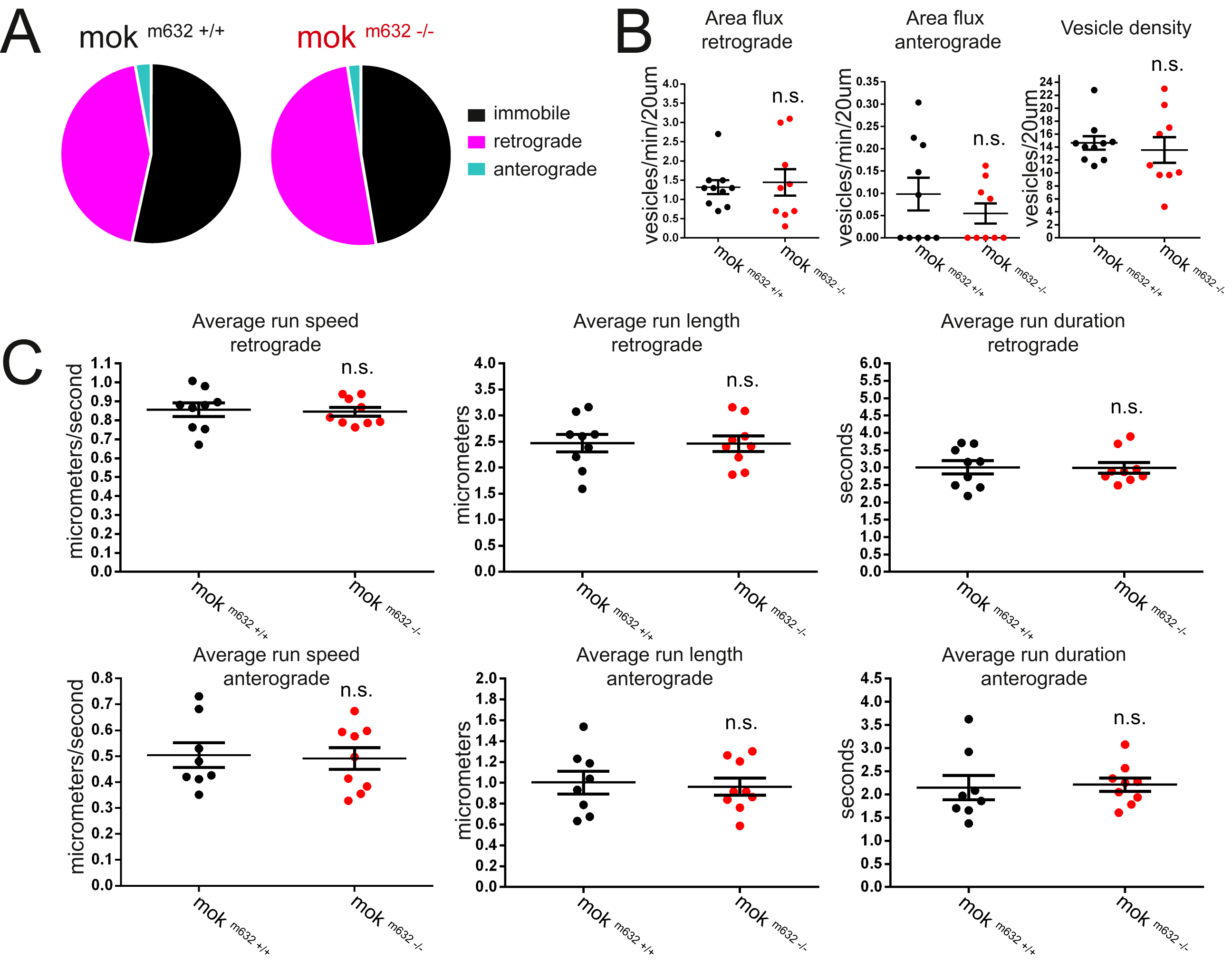

Supplement: Supplementary file 6 — Figure S6. Transport of p75 receptor (ngfra) is not affected by loss of Dynactin1a. In vivo timelapse imaging of vesicles tagged with ngfra-eGFP fusion protein show no changes in a) transport states, b) area flux and vesicle density, c) average run speed, length and duration of retrograde and anterograde runs of the trophic receptor in 2dpf CaP motor neurons. All data presented as average +/−SEM. (n kymogram = 10,9). (TIF 16813 kb) [file 13024_2019_327_MOESM6_ESM.tif]

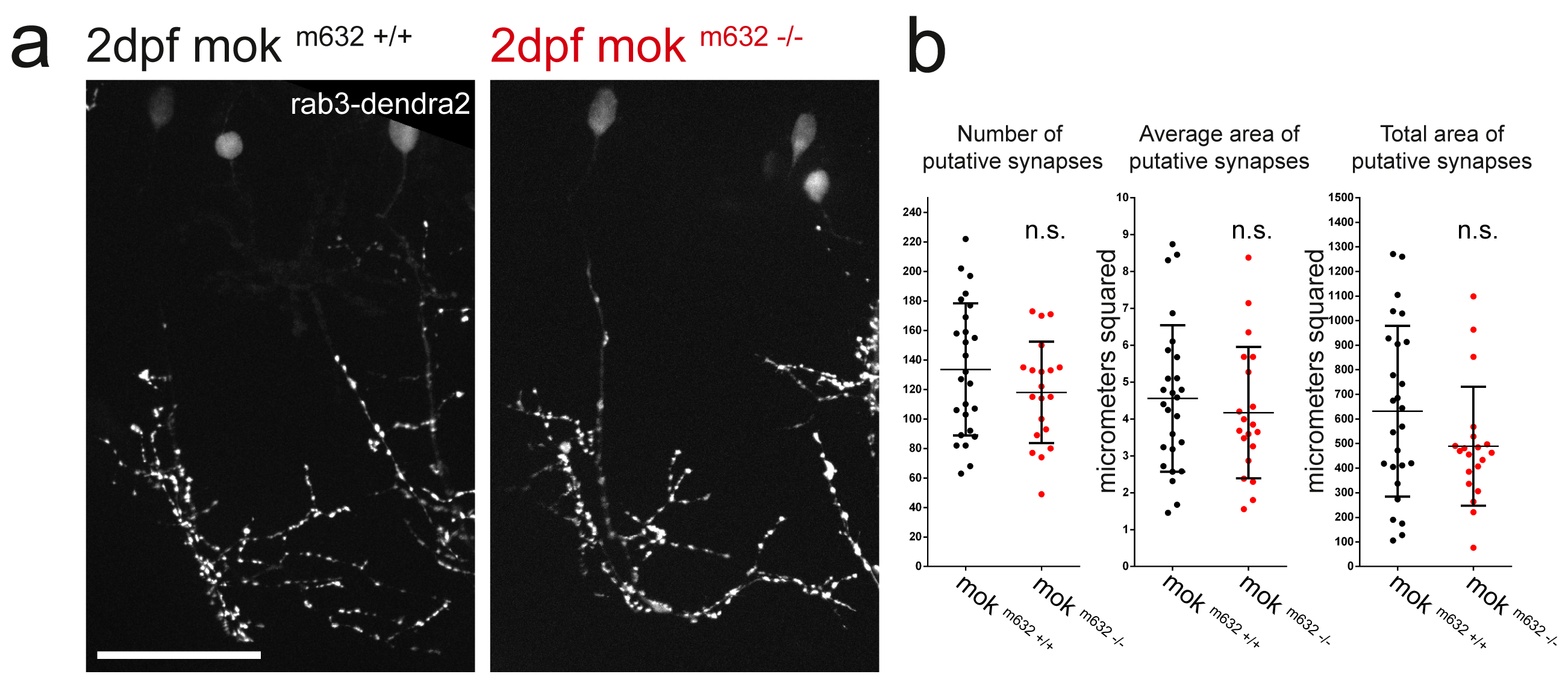

Supplement: Supplementary file 7 — Figure S7. Synapse distribution and size at 2dpf is not affected by loss of Dynactin1a. a) Putative synapses are visualized with rab3-dendra2 labeling in single CaP cells at 2dpf. b) Distribution is not affected, as determined by number, average area and total area, of putative synapses. All data shown as average +/− SD (b: n cells = 26,20). (TIF 12772 kb) [file 13024_2019_327_MOESM7_ESM.tif]

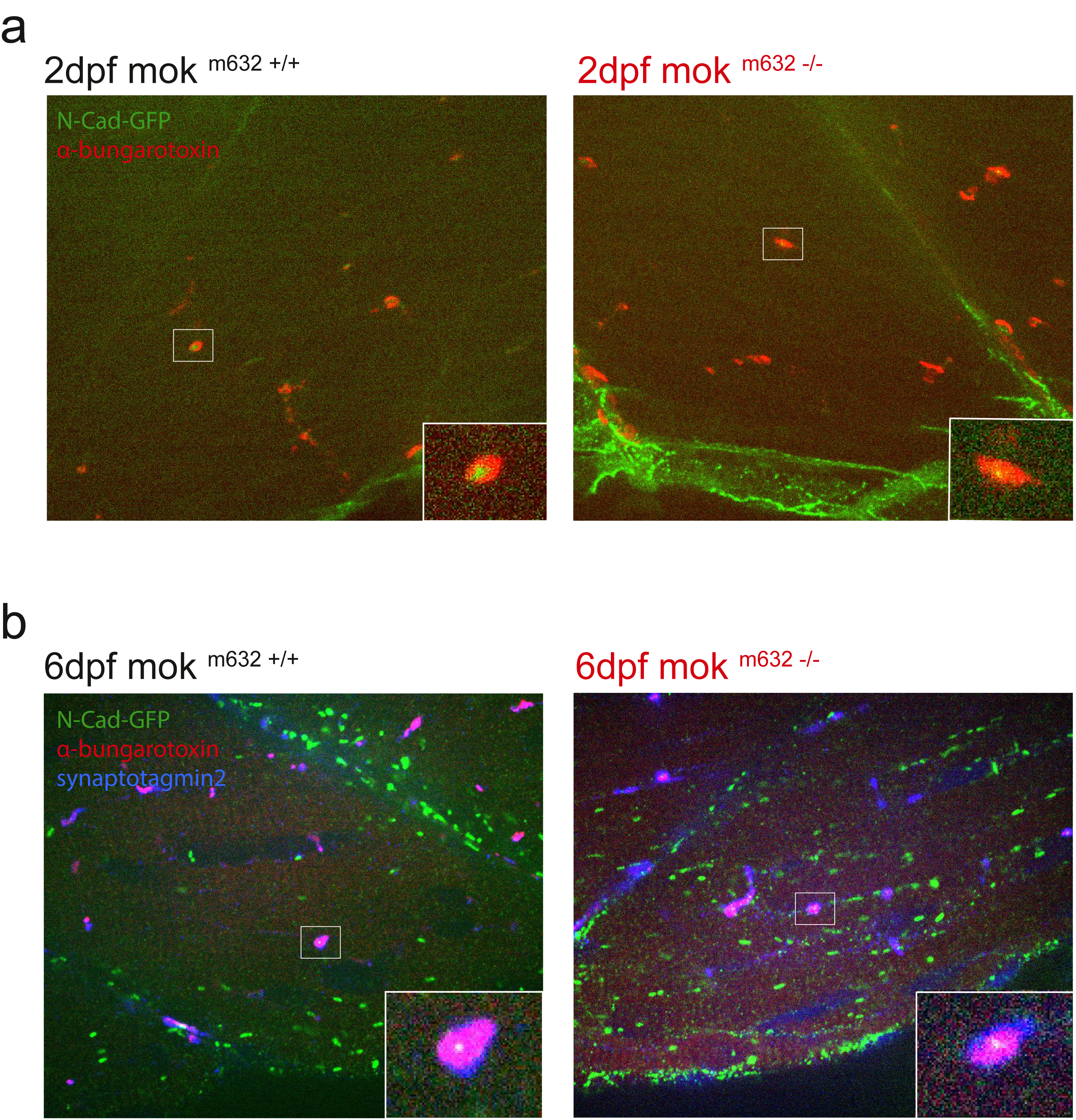

Supplement: Supplementary file 8 — Figure S8. N-Cadherin localization is not altered at mokm632−/− adherens junctions of the NMJ. a) Confocal projection of live 2dpf Tg(cdh2:Cdh2-GFP; mokm632; mnx1:Gal4) embryos showing N-Cadherin-GFP located at the center of a CaP NMJ synapse, determined by presence of post-synaptic AChR labelled with conjugated α-bungarotoxin (bath application, in red). b) Whole-mount immunohistochemistry of 6dpf Tg(cdh2:Cdh2-GFP; mokm632; mnx1:Gal4) larvae showing N-Cadherin-GFP (anti-GFP, in green) located at the center of an NMJ synapse, which was co-labeled to show pre-and postsynaptic structures (respectively, synaptotagmin2 in red and conjugated α-bungarotoxin in blue). Boxes show close-up of the synaptic structures, surrounding the N-Cadherin puncta. (TIF 40868 kb) [file 13024_2019_327_MOESM8_ESM.tif]

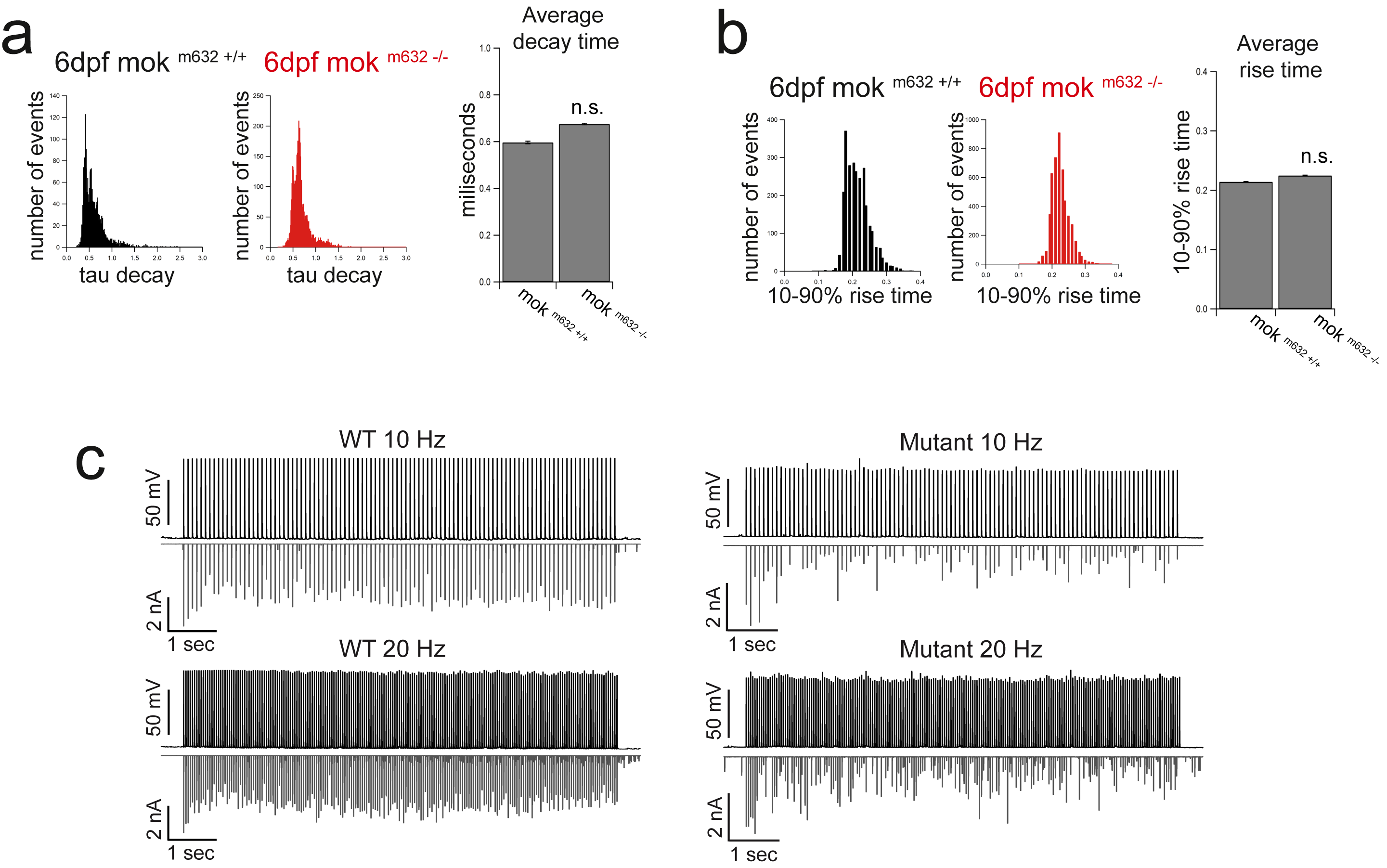

Supplement: Supplementary file 9 — Figure S9. Loss of Dynactin1a leads to abnormal physiological properties of the NMJ. Additional metrics of NMJ whole-cell recordings showing no change in a) Tau decay and b) rise time of 6dpf mEPC recordings in mokm632−/− larvae. c) Additional frequencies for paired-recordings of CaP-fast twitch muscle fibers at 6dpf showing similar failure rates of mutant NMJs. (TIF 18204 kb) [file 13024_2019_327_MOESM9_ESM.tif]

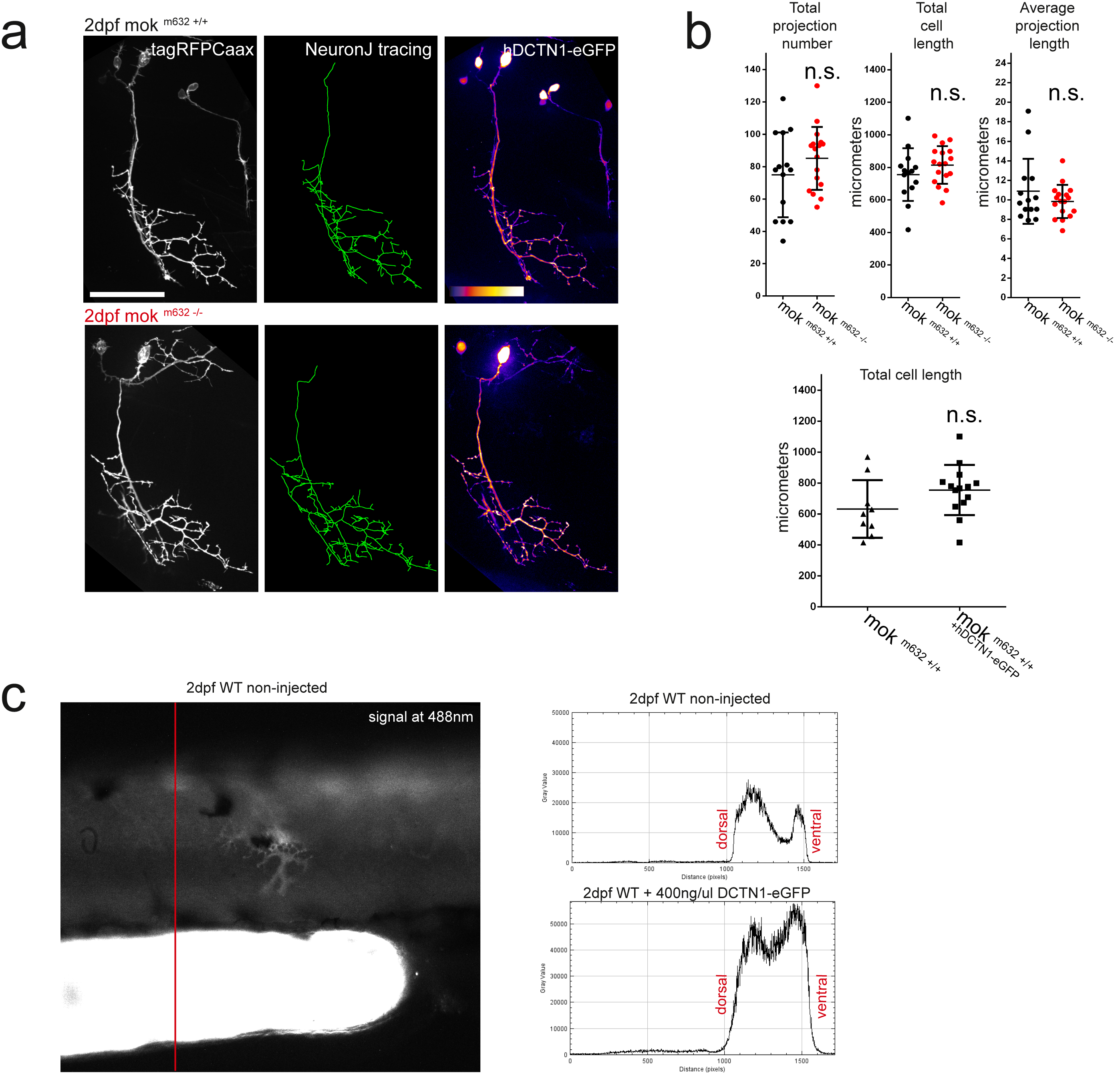

Supplement: Supplementary file 10 — Figure S10. Overexpression of human wild-type DCTN1 does not affect initial development of CaP motor neurons and exogenous expression is confirmed by increase in GFP signal. a) Overexpression of human wild-type DCTN1-GFP (heatmap), along with a membrane-bound reporter (tagRFP-Caax, traced in green), is obtained in a cell-autonomous manner and does not accumulate at synapses. b) Overexpression does not affect initial growth of CaP motor neurons in either mutant embryos or their wild-type siblings at 2dpf, as determined by total projection number, total cell length and average projection length. c) DCTN1-GFP RNA expression is confirmed by GFP detection in 2dpf embryos by fluorescent microscopy in the 488 nm channel, image shown here for non-injected embryos with red bar for histogram generation. Quantification confirms higher fluorescent signal in injected (400 ng/ul DCTN1-GFP) vs non injected wild-type embryos. All data shown as average +/− SD (b: n = 8,14; c: n = 10,5) (TIF 27508 kb) [file 13024_2019_327_MOESM10_ESM.tif]
